# Active Treatment vs Expectant Management of Patent Ductus Arteriosus in Preterm Infants: A Meta-Analysis

**DOI:** 10.1001/jamapediatrics.2025.1025

**Published:** 2025-05-27

**Authors:** Santosi Buvaneswarran, Yi Ling Wong, Shen Liang, Swee Chye Quek, Jiun Lee

**Affiliations:** 1 Department of Neonatology, Khoo Teck Puat National University Children’s Medical Institute, National University Hospital, National University Health System, Singapore; 2 Department of Pediatrics, Yong Loo Lin School of Medicine, National University of Singapore, Singapore; 3 Biostatistics Unit, Yong Loo Lin School of Medicine, National University of Singapore, Singapore; 4 Department of Paediatrics, Khoo Teck Puat National University Children’s Medical Institute, National University Hospital, National University Health System, Singapore

## Abstract

**Importance:**

Recent evidence suggests that expectant management of hemodynamically significant patent ductus arteriosus (PDA) may confer better outcomes.

**Objective:**

To assess clinical outcomes of active treatment vs expectant management of hemodynamically significant PDA in preterm infants.

**Data Sources:**

The PubMed (MEDLINE), Embase, and Cochrane Library databases were searched for randomized clinical trials (RCTs) published between January 1, 2010, and July 31, 2024.

**Study Selection:**

RCTs were included if they enrolled preterm infants born before 33 weeks of gestation and had 2 groups comparing active treatment with expectant management.

**Data Extraction and Synthesis:**

The Preferred Reporting Items for Systematic Reviews and Meta-Analyses statement was followed. Data were extracted by 2 independent reviewers. The Cochrane Risk of Bias tool was used to assess study quality. A random-effects model was used to estimate the pooled effects.

**Main Outcomes and Measures:**

The primary outcomes were a composite of death at 36 weeks’ postmenstrual age or at discharge (whichever occurred later) or moderate to severe bronchopulmonary dysplasia (BPD), death at 36 weeks, and moderate to severe BPD. Secondary outcomes included intraventricular hemorrhage, periventricular leukomalacia (PVL), retinopathy of prematurity, and necrotizing enterocolitis.

**Results:**

This meta-analysis included 10 RCTs involving 2035 infants. There were 1018 infants in the active treatment group (510 female [50.1%]) and 1017 infants (551 male [54.2%]) in the expectant management group. The mean (SD) gestational age was 26.2 (1.7) weeks vs 26.3 (1.7) weeks, and the mean (SD) birth weight was 874.7 (222.1) g vs 897.7 (216.5) g, respectively. The incidence of the composite outcome (death at 36 weeks or at discharge or moderate to severe BPD) was higher in the active treatment group vs the expectant management group (516 of 918 [56.2%] vs 465 of 915 [50.8%]; relative risk [RR], 1.10 [95% CI, 1.01-1.19]; *P* = .02). Deaths at 36 weeks were higher in the active treatment group (139 of 974 [14.3%] vs 109 of 969 [11.2%]; RR, 1.27 [95% CI, 1.01-1.61]; *P* = .04). There were nonsignificant increases in moderate to severe BPD (372 of 785 [47.4%] vs 349 of 806 [43.3%]; RR, 1.08 [95% CI, 0.95-1.23]; *P* = .25) and PVL (52 of 913 [5.7%] vs 32 of 908 [3.5%]; RR, 1.50 [95% CI, 0.98-2.30]; *P* = .06) in the active treatment group vs the expectant management group.

**Conclusions and Relevance:**

This meta-analysis found that active treatment of hemodynamically significant PDA during the first 2 weeks of life was associated with a significantly higher incidence of death or moderate to severe BPD and with increased mortality compared with an expectant management approach. Further research is needed for revision of protocols for PDA management.

## Key Points

**Question:**

Is active treatment of hemodynamically significant patent ductus arteriosus (PDA) in preterm infants better than an expectant management approach?

**Findings:**

This meta-analysis included 10 randomized clinical trials involving 2035 preterm infants born before 33 weeks of gestation. Compared with expectant management, active closure of hemodynamically significant PDA during the first 2 weeks of life was associated with potential harm, including significantly higher rates of mortality (16% vs 12%) and a significantly worse composite outcome of death at 36 weeks’ postmenstrual age or at discharge or moderate to severe bronchopulmonary dysplasia (56% vs 51%).

**Meaning:**

These findings suggest that an expectant management approach to PDA in preterm infants may be associated with a better morbidity and mortality profile.

## Teaser

This meta-analysis assesses clinical outcomes of active treatment vs expectant management of hemodynamically significant patent ductus arteriosus in preterm infants born before 33 weeks of gestation.

## Introduction

For decades, the conventional management approach to hemodynamically significant patent ductus arteriosus (PDA) in preterm infants has been to attempt closure with prostaglandin (PG) inhibitors, failing which surgical ligation is usually carried out.^1,2^ The rationale for PDA closure is to avoid cardiac failure and pulmonary overperfusion from a left-to-right shunt, which may minimize various PDA-associated morbidities, such as intraventricular hemorrhage (IVH), necrotizing enterocolitis (NEC), and bronchopulmonary dysplasia (BPD), for which PDA is thought to be an important causative factor.

Since the 2000s, however, there has been a shift toward less aggressive treatment for PDA because despite some success with pharmacologic closure, no other benefit could be conclusively demonstrated.^3,4,5^ The pharmacologic agents used also have some serious adverse effects.^6^ In addition, rates of spontaneous PDA closure in infants with a birth weight of 1000 g or more were very high in previous studies.^7^ Surgical ligation of PDA is accompanied by substantial risks and does not guarantee a good outcome.^8,9,10^ Nevertheless, in the majority of neonatal intensive care units, infants with a birth weight of less than 1000 g are still being routinely treated for hemodynamically significant PDA, despite reports of noninferiority of an expectant management approach.^9,10,11^ Sung et al^9^ compared outcomes between 2 historical cohorts of infants with a gestational age (GA) of 23 to 26 weeks. During the earlier period, the protocol was mandatory PDA closure, with 82% of infants undergoing PDA ligation at a mean (SD) age of 12 (7) days. In the later period, no infant received indomethacin or underwent PDA ligation. Rates of BPD were notably higher in the mandatory PDA closure cohort.^9^

Randomized clinical trials (RCTs) comparing active treatment vs expectant management of PDA are challenging to conduct because there is often insufficient clinician equipoise, resulting in difficulty in patient recruitment. Sosenko et al^12^ and Kluckow et al^13^ were the first to conduct trials using an echocardiography-guided targeted approach to the treatment of the PDA. Clyman et al^14^ conducted the largest RCT of pharmacologic treatment vs expectant management of PDA for preterm infants. Their results did not show better outcomes for the active treatment group. Instead, active treatment demonstrated harm in a certain subgroup of infants.^14^ Two similar large trials were completed in 2023 and 2024.^15,16^ The results of these large trials could potentially affect the way we manage PDA in preterm infants. We therefore performed a meta-analysis of RCTs comparing active treatment vs expectant management of PDA.

## Methods

This meta-analysis was conducted using data from published studies where ethical approval and informed consent were obtained by the original investigators; therefore, no separate ethical approval was required. The study followed the Preferred Reporting Items for Systematic Reviews and Meta-Analyses (PRISMA) reporting guideline. The research protocol is registered in the International Prospective Register of Systematic Reviews (PROSPERO; CRD42024541379).

### Search Strategy

A literature search was conducted by 2 independent reviewers (S.B. and Y.L.W.) using the PubMed (MEDLINE), Embase, and Cochrane Library databases, including both MeSH (Medical Subject Headings) terms and related keywords. The detailed search strategy is available in eTable 1 in Supplement 1. The search was limited to studies that were published in English between January 1, 2010, and July 31, 2024, and included human participants only. We chose to limit the search to studies published from 2010 onward because antenatal steroid use for preterm delivery was then more prevalent and consistent.^17^ The reference lists of included articles were screened for additional articles.

### Selection Criteria

RCTs were included if they studied preterm infants born before 33 weeks of gestation with hemodynamically significant PDA (diagnosed by clinical or echocardiographic criteria) and had 2 groups comparing active treatment of PDA (pharmacologic, surgical ligation, percutaneous device closure) vs expectant management. Studies that administered treatment for the purpose of prophylaxis against PDA (ie, PDA was not determined to be present and hemodynamically significant before administration of the intervention) were excluded.^18^

Primary outcomes were as follows: (1) composite of death at 36 weeks’ postmenstrual age (hereinafter, 36 weeks) or at discharge (whichever occurred later) or moderate to severe BPD,^19,20^ (2) composite of death at 36 weeks or moderate to severe BPD, (3) death at 36 weeks, (4) death at 36 weeks or at discharge (whichever occurred later), and (5) moderate to severe BPD. Secondary outcomes included death before hospital discharge, death at 28 days, cause of death, IVH, periventricular leukomalacia (PVL), retinopathy of prematurity (stage 3) or requiring treatment, pulmonary hemorrhage, cardiovascular support (hypotension, inotropic support, or both), pulmonary hypertension, NEC (Bell stage ≥2), gastrointestinal perforation, gastrointestinal bleeding, time to full enteral feeding, diuretic use, sepsis, kidney failure, postnatal steroid use, surgical ligation, duration of respiratory support (oxygen vs invasive vs noninvasive), length of hospital stay, PDA status at discharge, weight gain, and discharge home with respiratory or oxygen support.

### Data Collection and Quality Assessment

Two authors (S.B. and Y.L.W.) independently conducted the search in the chosen databases using the predetermined search syntax, reviewed the abstracts, and reviewed the full text of the included articles. Any discrepancies were mutually resolved by consensus or in consultation with a third author (J.L.). Data collection was performed by 2 authors (S.B. and Y.L.W.) independently and verified by a third author (J.L.). Any discrepancies were mutually resolved. The Cochrane Risk of Bias tool was used to assess study quality.^21^

### Statistical Analysis

A random-effects model was chosen to estimate the pooled effects, including both relative risk (RR) and risk difference (RD). Heterogeneity among different studies was evaluated with the Cochran *Q* test and *I*^2^ statistics. Small-study effects were examined with the regression-based Egger test and the Begg test. Subgroup analyses were conducted for articles reporting on infants born before 29 weeks of gestation for the reported primary outcomes. Forest plots were generated to visualize the treatment effect of the individual studies and the pooled effect. *P* ≤ .05 (2-tailed) for RR was considered statistically significant. Stata, version 18 (StataCorp LLC), was used to perform the meta-analysis.

## Results

### Study Identification and Selection

**Figure 1.**
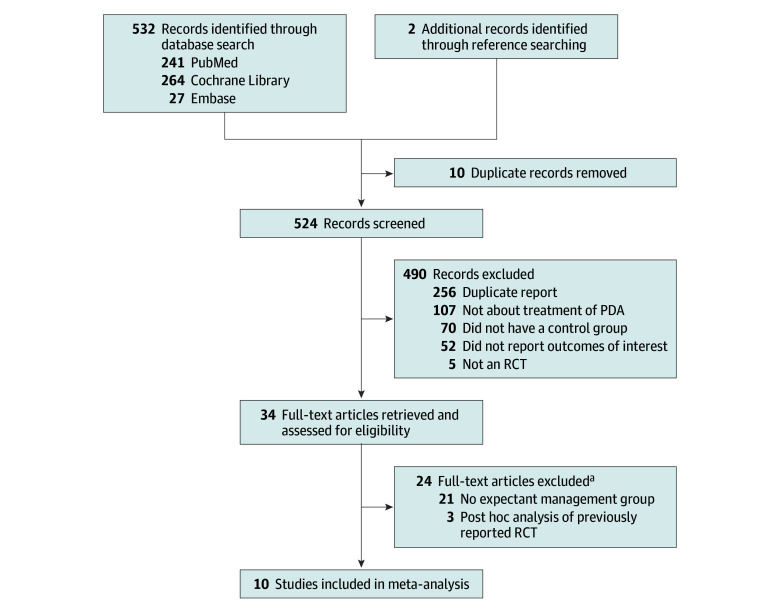
Study Flow Diagram of the Study Selection Process ^a^No full-text articles were excluded due to publication before 2010. PDA indicates patent ductus arteriosus; RCT, randomized clinical trial.

The search was conducted per the predefined search strategy. The full search results are reported in Figure 1. Of the 524 abstracts screened, 490 were excluded for the following reasons: (1) did not report predefined primary outcomes, (2) were not about treatment of PDA, (3) were not RCTs, (4) did not have a control group, or (5) were duplicates. A total of 34 articles underwent full-text review; 24 articles were excluded, 19 of which were published before 2010 but were excluded for reasons other than year of publication (eTable 2 in Supplement 1). Ten articles were included in this meta-analysis,^12,13,14,15,16,22,23,24,25,26^ all of which evaluated treatment of PDA in the first 2 weeks of life. A total of 6 studies used ibuprofen in their active treatment group,^12,15,16,22,23,26^ 1 study used indomethacin,^13^ and 3 studies used more than 1 medication (ibuprofen, indomethacin, or paracetamol).^14,24,25^ Details on the design of each study are provided in eTable 3 in Supplement 1. Secondary outcomes and BPD definitions are included in eTable 4 in Supplement 1. Differences between the PROSPERO protocol and our final meta-analysis are summarized in eTable 5 in Supplement 1.

### Baseline Characteristics

A total of 2035 infants were enrolled in the included trials: 1018 in the active treatment group (510 female [50.1%] and 508 male [49.9%]) and 1017 in the expectant management group (466 female [45.8%] and 551 male [54.2%]). eTable 6 in Supplement 1 describes the baseline characteristics of the study population. Infants in the active treatment group vs the expectant management group had a mean (SD) GA of 26.2 (1.7) weeks vs 26.3 (1.7) weeks and a mean (SD) birth weight of 874.7 (222.1) g vs 897.7 (216.5) g, respectively. Other baseline characteristics were similar between groups. Open-label medical treatment was administered to 164 patients (16.1%) in the active treatment group compared with 297 (29.2%) in the expectant management group. PDA treatment was started during the first 2 weeks of life in all 10 trials and within the first 72 hours of life (HOL) in 7 trials.^13,15,16,22,23,24,25^

### Risk-of-Bias Assessment

Risk of bias was assessed for all included studies, as illustrated in eFigure 1 in Supplement 1. Three studies were deemed to have low risk of bias.^16,24,26^ The other 7 studies were overall deemed as having some concerns for bias, mainly arising due to deviations from the intended intervention (eg, treatment of PDA in the expectant management group).^12,13,14,15,22,23,25^

### Primary Outcomes

The results of the primary outcomes are presented as RRs and RDs in Table 1. Forest plots for outcomes not described in the main text are presented in eFigure 2 in Supplement 1.

**Table 1.** Summary of Results for Primary Outcomes

Primary outcome	No./total No. (%) of patients		Risk difference, % (95% CI)	*P* value	Relative risk (95% CI)	*P* value
	Active treatment group (n = 1018)	Expectant management group (n = 1017)				
**Composite **						
Death at 36 wk PMA or at discharge (whichever occurred later) or moderate to severe BPD^a^	516/918 (56.2)	465/915 (50.8)	5.4 (1.1-9.7)	.01	1.10 (1.01- 1.19)	.02
GA <29 wk^b^	474/760 (62.4)	431/760 (56.7)	5.0 (−1.1 to 11.1)	.11	1.09 (1.01-1.18)	.03
Death at 36 wk PMA or moderate to severe BPD^a^	486/874 (55.6)	434/867 (50.1)	5.9 (1.2-10.6)	.01	1.10 (1.00-1.21)	.06
GA <29 wk	448/716 (62.6)	405/712 (56.9)	5.4 (−1.2 to 11.9)	.11	1.09 (0.99-1.21)	.12
**Noncomposite**						
Death at 36 wk PMA	139/974 (14.3)	109/969 (11.2)	2.8 (0.1-5.6)	.04	1.27 (1.01-1.61)	.04
GA <29 wk	113/746 (15.1)	84/742 (11.3)	3.9 (0.6-7.2)	.02	1.34 (1.03-1.75)	.03
Death at 36 wk PMA or at discharge (whichever occurred later)^a^	158/1018 (15.5)	126/1017 (12.4)	2.8 (−0.2 to 5.7)	.06	1.25 (1.01-1.56)	.04
GA <29 wk	128/790 (16.2)	95/790 (12.0)	3.9 (0.6-7.3)	.02	1.35 (1.05-1.73)	.02
Moderate to severe BPD	372/785 (47.4)	349/806 (43.3)	4.5 (−0.8 to 9.8)	.09	1.08 (0.95-1.23)	.25
GA <29 wk	354/647 (54.7)	340/671 (50.7)	3.1 (−4.2 to 10.4)	.41	1.07 (0.93-1.22)	.36

Abbreviation: BPD, bronchopulmonary dysplasia; GA, gestational age; PMA, postmenstrual age.

^a^ Post hoc outcomes (refer to eTable 5 in Supplement 1).

^b^ Subgroup analysis of infants with a GA less than 29 weeks.

#### Composite of Death or BPD

**Figure 2.**
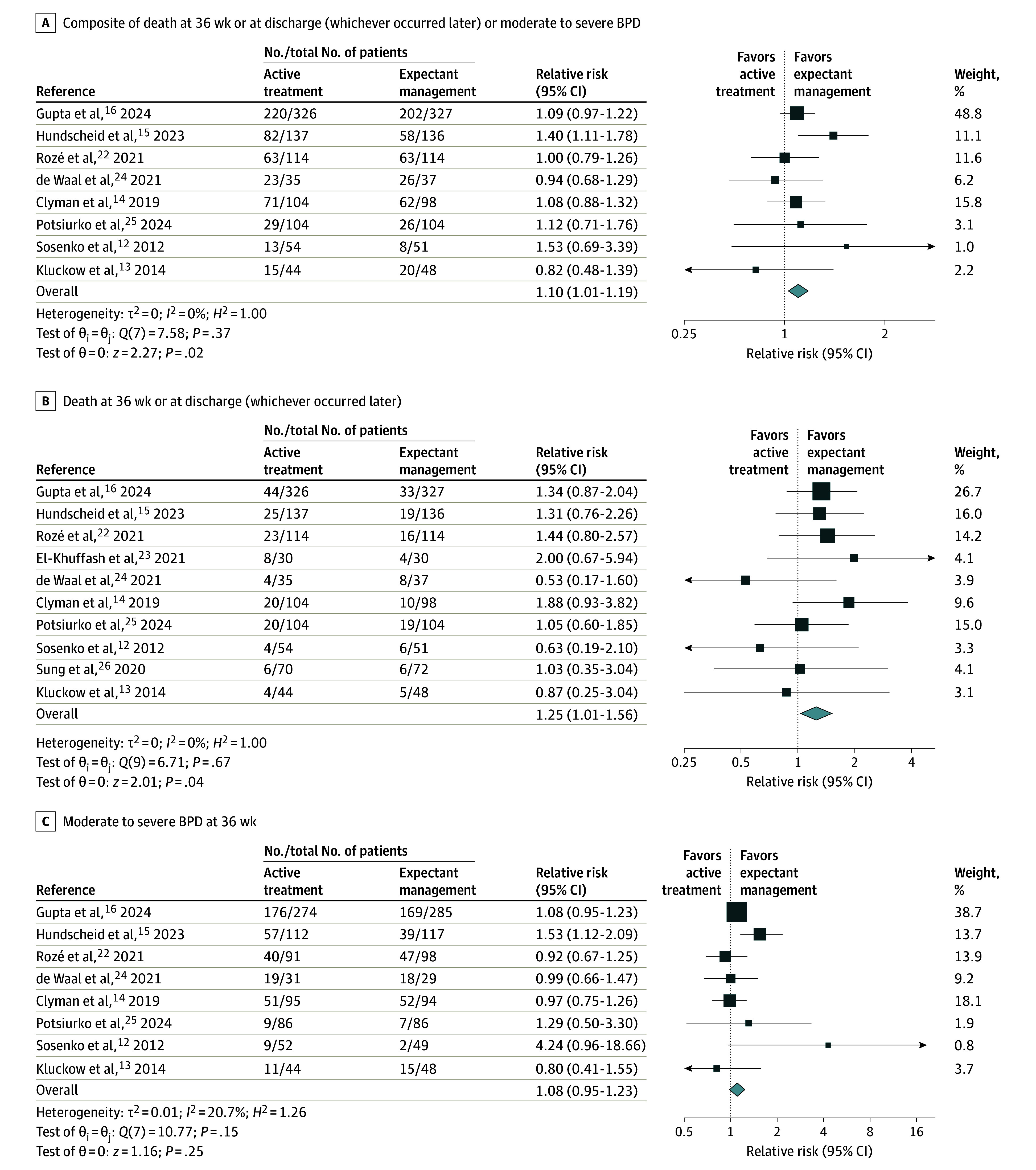
Forest Plots for Key Outcomes A random-effects restricted maximum likelihood model was used. In each panel, the sizes of the squares reflect the weight of each study, and the width of the diamond represents the 95% CI for the point estimate of the pooled effect. BPD indicates bronchopulmonary dysplasia.

Eight studies (n = 1833) reported a significantly higher incidence of the composite outcome of death at 36 weeks or at discharge (whichever occurred later) or moderate to severe BPD in the active treatment group vs the expectant management group (516 of 918 [56.2%] vs 465 of 915 [50.8%]; RD, 5.4% [95% CI, 1.1%- 9.7%]; RR, 1.10 [95% CI, 1.01-1.19]; *P* = .02) (Table 1 and Figure 2A).^12,13,14,15,16,22,24,25^ Subgroup analysis for the composite outcome for infants with a GA less than 29 weeks yielded similar results for the active treatment group vs the expectant management group (474 of 760 [62.4%] vs 431 of 760 [56.7%]; RD, 5.0% [95% CI, −1.1% to 11.1%]; RR, 1.09 [95% CI, 1.01-1.18]; *P* = .03) (Table 1 and eFigure 3 in Supplement 1).

Additional subgroup analyses were conducted for articles reporting on infants born before 26 weeks of gestation, mode of ventilation at time of randomization (noninvasive vs invasive), HOL at treatment (≤72 HOL vs >72 HOL), and medication type (ibuprofen vs indomethacin vs >1 agent) for the composite outcome of death at 36 weeks or at discharge (whichever occurred later) or moderate to severe BPD. No statistically significant differences were observed (eFigure 4 in Supplement 1).

Seven studies (n = 1741) reported a nonsignificant increase in death at 36 weeks or moderate to severe BPD in the active treatment group vs in the expectant management group (486 of 874 [55.6%] vs 434 of 867 [50.1%]; RD, 5.9% [95% CI, 1.2%-10.6%]; RR, 1.10 [95% CI, 1.00-1.21]; *P* = .06) (Table 1 and eFigure 2 in Supplement 1).^12,14,15,16,22,24,25^

#### Death

Nine studies (n = 1943) reported significantly more deaths at 36 weeks in the active treatment group vs the expectant management group (139 of 974 [14.3%] vs 109 of 969 [11.2%]; RD, 2.8% [95% CI, 0.1%-5.6%]; RR, 1.27 [95% CI, 1.01-1.61]; *P* = .04) (Table 1 and eFigure 5 in Supplement 1).^12,14,15,16,22,23,24,25,26^ All 10 studies (n = 2035) reported more deaths at 36 weeks or at discharge (whichever occurred later) in the active treatment group vs the expectant management group (158 of 1018 [15.5%] vs 126 of 1017 [12.4%]; RD, 2.8% [95% CI, −0.2% to 5.7%]; RR, 1.25 [95% CI, 1.01-1.56]; *P* = .04) (Table 1 and Figure 2B).^12,13,14,15,16,22,23,24,25,26^ For subgroups of infants with a GA less than 29 weeks, deaths (both definitions) were also significantly higher in the active treatment group compared with the expectant management group (Table 1 and eFigures 6 and 7 in Supplement 1).

#### BPD

Eight studies (n = 1833) reported on moderate to severe BPD, which showed a nonsignificant increase in the active treatment group vs the expectant management group (372 of 785 [47.4%] vs 349 of 806 [43.3%]; RD, 4.5% [95% CI, −0.8% to 9.8%]; RR, 1.08 [95% CI, 0.95-1.23]; *P* = .25) (Table 1 and Figure 2C).^12,13,14,15,16,22,24,25^ The effect size was similar for the subgroup analysis (6 studies [n = 1520]) of infants with a GA less than 29 weeks (354 of 647 [54.7%] vs 340 of 671 [50.7%]; RD, 3.1% [95% CI, −4.2% to 10.4%]; RR, 1.07 [95% CI, 0.93-1.22]; *P* = .36) (Table 1 and eFigure 8 in Supplement 1).^13,14,15,16,22,24^

### Secondary Outcomes

Results of the analysis of secondary outcomes in RR and RD with the relevant *P* values are presented in Table 2. Eight studies (n = 1821) showed a nonsignificant increase in PVL in the active treatment group vs the expectant management group (52 of 913 [5.7%] vs 32 of 908 [3.5%]; RR, 1.50 [95% CI, 0.98-2.30]; *P* = .06).^12,13,14,15,16,22,23,25^ However, there was a significant risk difference for PVL between the 2 groups, favoring expectant management (RD, 1.8% [95% CI, 0.4%-3.2%]; *P* = .01). There were no statistically significant differences between groups for the other listed secondary outcomes. Not all trials in this meta-analysis reported cause of death. There was a slight preponderance of sepsis and gastrointestinal causes of death in the active treatment group. The reported causes of death are presented in eTables 7 and 8 in Supplement 1.

**Table 2.** Summary of Results for Secondary Outcomes

Secondary outcome	No./total No. (%) of patients		Risk difference, % (95% CI)	*P* value	Relative risk (95% CI)	*P* value
	Active treatment group (n = 1018)	Expectant management group (n = 1017)				
Death before discharge	62/406 (15.3)	50/403 (12.4)	2.0 (−2.5 to 6.5)	.38	1.22 (0.86-1.73)	.26
Grade III or IV IVH with ventricular dilation or intraparenchymal abnormality	119/974 (12.2)	96/969 (9.9)	1.2 (−1.3 to 3.8)	.35	1.24 (0.96-1.60)	.10
Periventricular leukomalacia	52/913 (5.7)	32/908 (3.5)	1.8 (0.4-3.2)	.01	1.50 (0.98-2.30)	.06
Retinopathy of prematurity						
Stage >2	81/560 (14.5)	77/563 (13.7)	−0.1 (−3.5 to 3.3)	.97	1.06 (0.80-1.41)	.68
Treatment	85/650 (13.1)	85/655 (13.0)	−1.1 (−4.5 to 2.3)	.53	1.01 (0.76-1.34)	.94
Pulmonary hemorrhage	57/864 (6.6)	63/864 (7.3)	−1.3 (−3.9 to 1.4)	.36	0.81 (0.46-1.42)	.46
Hypotension, inotropic or vasopressor support, or fluid expansion	283/859 (3.3)	286/857 (3.3)	−1.0 (−5.3 to 3.4)	.66	1.00 (0.88-1.14)	.99
Kidney failure	58/469 (12.4)	48/474 (10.1)	1.6 (−1.6 to 4.8)	.33	1.23 (0.86-1.75)	.26
Blood culture positive for sepsis	277/983 (28.2)	252/980 (25.7)	2.6 (−0.9 to 6.1)	.14	1.06 (0.92-1.22)	.42
Necrotizing enterocolitis stage ≥2	105/934 (11.2)	103/936 (11.0)	0.6 (−2.0 to 3.2)	.64	1.00 (0.77-1.29)	.98
Intestinal perforation	49/710 (6.9)	40/713 (5.6)	0.2 (−0.7 to 1.2)	.65	1.20 (0.80-1.81)	.37
Gastrointestinal bleeding	24/646 (3.7)	22/652 (3.4)	NA	NA	1.05 (0.60-1.85)	.86
PDA surgical ligation	47/1018 (4.6)	76/1017 (7.5)	NA	NA	0.67 (0.42-1.06)	.08
Postnatal steroids	207/730 (28.4)	188/727 (25.9)	3.1 (−1.6 to 7.9)	.20	1.11 (0.88-1.40)	.39

Abbreviations: IVH, intraventricular hemorrhage; NA, not applicable; PDA, patent ductus arteriosus.

eFigures 2, 9, and 10 in Supplement 1 are forest plots for the other primary or secondary outcomes for which a meta-analysis was conducted. eTable 9 in Supplement 1 presents the secondary outcomes for which only a review was conducted due to insufficient data to perform a meta-analysis.

## Discussion

The findings of this meta-analysis suggest that active closure of PDA during the first 2 weeks of life may potentially be harmful for preterm infants with a GA less than 33 weeks. Death at 36 weeks or later was significantly higher in the active treatment group vs the expectant management group (158 of 1018 [15.5%] vs 126 of 1017 [12.4%]; *P* = .04) and for the composite outcome of death at 36 weeks or later or moderate to severe BPD (516 of 918 [56.2%] vs 465 of 915 [50.8%]; *P* = .02). For the subgroup of infants with a GA less than 29 weeks, poorer outcomes of similar magnitudes were seen. There was a nonsignificant increase in moderate to severe BPD in the active treatment group. In a European population-based cohort study, PDA treatment was associated with a notably higher adjusted risk of BPD or death.^10^ In a previous study that used Finnish national registry data for preterm infants, Härkin et al^27^ found an association between mortality and PDA treatment after adjustment for known risk factors. In an observational study on extremely preterm infants, lower rates of PDA treatment were associated with a decrease in mortality.^28^

Not all trials reported cause of death. Available data for cause of death are presented in eTables 7 and 8 in Supplement 1. There was a slight preponderance of sepsis and gastrointestinal causes of death in the active treatment group. Based on our findings, it was not possible to explain the cause of the higher mortality rates seen in the active treatment group.

There could be 2 reasons that explain the better outcomes seen in the expectant management group: (1) PDA could confer physiologic advantages and survival protection for the preterm infant and/or (2) the pharmacologic agents used may have deleterious effects of their own.^29^ In preterm infants with respiratory distress syndrome (RDS), pulmonary blood flow (PBF) was substantially lower in those with more severe lung disease. As lung disease worsened, the PDA augmented PBF, thereby improving left ventricular output (LVO), resulting in better cerebral blood flow (CBF).^30,31^ Impaired CBF could explain our observation that PVL incidence was higher in the active treatment group. Ductal patency resulted in higher systemic blood flow compared with when the ductus was ligated. Previous studies have reported that ductal ligation led to left ventricular dysfunction and lower LVO.^30,32^ Arterial partial pressure of oxygen (Pao_2_) was substantially higher and partial pressure of carbon dioxide was notably lower when the ductus was open.^33^ Tooley^34^ listed pulmonary ischemia as a major factor in the pathogenesis of hyaline membrane disease. In that study, pulmonary ischemia caused impaired cellular metabolism and surfactant synthesis, and it reduced lung volume and compliance. Pulmonary vasodilation with acetylcholine improved arterial Pao_2_ and lung compliance.^34^ With lung ischemia, the ensuing inflammatory cascade worsened lung injury.^35,36^ Thus, PDA could be a beneficial adaptive response in preterm infants with lung disease to increase pulmonary and CBF and improve oxygenation. Moreover, in preterm infants with lung disease and pulmonary hypertension, PDA may serve as a pop-off valve to reduce right ventricular afterload, minimizing risk of cor pulmonale.^37^

PDA is more common in infants with lung disease, both in RDS and in BPD.^27,38,39^ It is likely that the ductus was patent secondary to the presence of lung disease, rather than the PDA itself causing more severe lung disease.^27^ In RDS, PG and inflammatory cytokine levels were high.^40,41^ BPD severity was also directly correlated with the degree of inflammation.^42^ Proinflammatory cytokines in turn stimulate cyclooxygenase COX-2 and PG synthesis.^43^ Potent vasodilatory cytokines (eg, tumor necrosis factor-α, interleukin-6) maintain ductal patency independent of PG pathways.^44,45^ COX inhibitors, peroxidase inhibitors, and corticosteroids constrict the ductus by reducing PG and inflammatory cytokine levels.^46,47,48,49^ COX inhibitor success rates of only 13% to 56% are likely due to the unopposed vasodilatory effects of cytokines.^11^ In previous studies, the observation that PDAs closed as BPD improved in severity, or after an infant completed a course of postnatal steroids, is consistent with the role of inflammation in maintaining ductal patency.^48,50^

Indomethacin, ibuprofen, and paracetamol are commonly used to close PDA. Indomethacin reduces CBF and oxygenation in preterm infants.^51,52^ In the preterm lung, indomethacin has been reported to impair surfactant production, reduce lymphangiogenesis, and increase the fraction of inspired oxygen requirement.^53,54,55^ Previous studies have reported that ibuprofen had substantial pulmonary vasoconstrictive effects and inhibited newborn lung angiogenesis.^56,57,58^ Paracetamol metabolism via the cytochrome P450 2E1 pathway produces the toxic metabolite *N*-acetyl-*p*-benzoquinone imine, which covalently binds to intracellular proteins, causing cellular dysfunction and death.^59^ Paracetamol increases pulmonary vascular resistance, and its use in preterm infants for PDA treatment has been associated with increased mortality.^60,61^ All 3 medications affected the preterm lung, which could lead to increased hypoxemia.

Controversy exists regarding the definition of hemodynamically significant PDA. The commonly used criterion is PDA diameter. In a previous study, a PDA diameter larger than 1.5 mm at days 1 to 2 of life was associated with the need for later treatment of symptomatic PDA.^62^ However, there was no reduction in PDA-associated morbidities, despite pharmacologic treatment targeted at infants with hemodynamically significant PDAs using diameter as a proxy for PDA severity.^62^ A PDA severity score has been devised in an attempt to better select infants with truly hemodynamically significant PDA. In previous studies, the proportion of infants meeting treatment criteria using the PDA severity score (approximately 50%) was similar to that when PDA diameter was the criterion for hemodynamically significant PDA (approximately 33%).^16,63^ Although PDA is the result of substantial preterm lung disease and not the cause of it, its presence still needs to be managed to avoid pulmonary overperfusion. Management can be achieved without nonsteroidal anti-inflammatory drugs (NSAIDs) or paracetamol by optimal ventilation, judicious fluids, and prudent use of diuretics.

### Strengths and Limitations

The sample size of the RCTs included in this meta-analysis was large, with 2035 infants total. There was also minimal study heterogeneity, suggesting insignificant variation in the true treatment effect size.

This meta-analysis has some limitations. Recruitment included infants from a wide range of GAs, and studies used different PDA treatment strategies with different pharmacologic agents. Despite this, the benefits of expectant management were also seen in the subgroup of more immature infants (<29 weeks of GA) regardless of treatment protocol. Because PDA treatment was started during the first 2 weeks of life (7 trials started within the first 72 HOL), our results may not be generalizable to infants who received later treatment. Open-label medical treatment was greater in the expectant management group (297 [29.2%]) vs the active treatment group (164 [16.1%]). Overall, lesser use of NSAIDs or paracetamol and more prolonged PDA exposure with expectant management was associated with improved outcomes. BPD definitions varied among the RCTs, although most carried out the molecular oxygen reduction test devised by Walsh et al (defining moderate to severe BPD).^19,20,64^ Subgroup analyses of other clinical points of interest (eFigure 4 in Supplement 1) did not show any significant differences for the composite outcome of death at 36 weeks or at discharge (whichever occurred later) or moderate to severe BPD. However, we were unable to rule out a type II error (false negative) due to the small number of studies reporting these data.

## Conclusions

The findings of this meta-analysis appear to challenge the time-honored conventional wisdom in the management of PDA in preterm infants. Expectant management in the first 2 weeks of life was associated with significant reductions in mortality and in the composite outcome of death at 36 weeks or at discharge or moderate to severe BPD. Our results signal a need to review existing protocols for aggressively closing PDA. Until new data emerge from ongoing trials, the use of NSAIDs or paracetamol during the first 2 weeks of life for PDA closure must be undertaken cautiously, balancing presumed benefits and potential risks.^65,66^

## Notes

Supplement 1. **eTable 1.** Search Strategy **eTable 2.** Excluded Full-Text Articles (n = 24) and Reasons for Their Exclusion **eTable 3.** Individual Article Characteristics **eTable 4.** Individual Articles’ Secondary Outcomes and Bronchopulmonary Dysplasia (BPD) Definition **eTable 5.** Differences Between A Priori PROSPERO Protocol and Final Study **eTable 6.** Baseline Characteristics **eFigure 1.** Risk of Bias Assessment **eFigure 2.** Forest Plots for Primary and Secondary Outcomes Not Demonstrated in the Main Article **eFigure 3.** Forest Plot Showing Subgroup Analysis at Less Than 29 Weeks for Composite Primary Outcome: Death at 36 Weeks or at Discharge (Whichever Occurred Later) or Moderate to Severe Bronchopulmonary Dysplasia in Figure 2A **eFigure 4.** Forest Plots of Additional Subgroup Analyses **eFigure 5.** Forest Plot Showing Death at 36 Weeks **eFigure 6.** Forest Plot Showing Subgroup Analysis at Less Than 29 Weeks for Outcome: Death at 36 Weeks in eFigure 5 **eFigure 7.** Forest Plot Showing Subgroup Analysis at Less Than 29 Weeks for Outcome: Death at 36 weeks or at Discharge (Whichever Occurred Later) in Figure 2B **eFigure 8.** Forest Plot Showing Subgroup Analysis at Less Than 29 Weeks: Moderate to Severe Bronchopulmonary Dysplasia at 36 Weeks in Figure 2C **eTable 7.** Cause of Death **eTable 8.** Cause of Death (Grouped by System) **eFigure 9.** Forest Plot Showing Bronchopulmonary Dysplasia at 36 Weeks **eFigure 10.** Forest Plot showing Subgroup Analysis at Less Than 29 Weeks for Outcome: Bronchopulmonary Dysplasia (BPD) at 36 Weeks in eFigure 9 **eTable 9.** Secondary Outcomes for Which Meta-Analysis Was Not Done
eReferences

Supplement 2.
Data Sharing Statement

## References

[1] Heymann MA, Rudolph AM, Silverman NH. Closure of the ductus arteriosus in premature infants by inhibition of prostaglandin synthesis. N Engl J Med. 1976;295(10):530-533. doi:10.1056/NEJM197609022951004 950959

[2] Gersony WM, Peckham GJ, Ellison RC, Miettinen OS, Nadas AS. Effects of indomethacin in premature infants with patent ductus arteriosus: results of a national collaborative study. J Pediatr. 1983;102(6):895-906. doi:10.1016/S0022-3476(83)80022-5 6343572

[3] Bixler GM, Powers GC, Clark RH, Walker MW, Tolia VN. Changes in the diagnosis and management of patent ductus arteriosus from 2006 to 2015 in United States neonatal intensive care units. J Pediatr. 2017;189:105-112. doi:10.1016/j.jpeds.2017.05.024 28600155

[4] Benitz WE; Committee on Fetus and Newborn. Patent ductus arteriosus in preterm infants. Pediatrics. 2016;137(1):e20153730. doi:10.1542/peds.2015-3730 26672023

[5] Mitra S, Scrivens A, von Kursell AM, Disher T. Early treatment versus expectant management of hemodynamically significant patent ductus arteriosus for preterm infants. Cochrane Database Syst Rev. 2020;12(12):CD013278. 33301630 10.1002/14651858.CD013278.pub2 PMC8812277

[6] Sivanandan S, Agarwal R. Pharmacological closure of patent ductus arteriosus: selecting the agent and route of administration. Paediatr Drugs. 2016;18(2):123-138. doi:10.1007/s40272-016-0165-5 26951240

[7] Nemerofsky SL, Parravicini E, Bateman D, Kleinman C, Polin RA, Lorenz JM. The ductus arteriosus rarely requires treatment in infants >1000 grams. Am J Perinatol. 2008;25(10):661-666. doi:10.1055/s-0028-1090594 18850514

[8] Clyman R, Cassady G, Kirklin JK, Collins M, Philips JB III. The role of patent ductus arteriosus ligation in bronchopulmonary dysplasia: reexamining a randomized controlled trial. J Pediatr. 2009;154(6):873-876. doi:10.1016/j.jpeds.2009.01.005 19324366 PMC2709418

[9] Sung SI, Chang YS, Chun JY, . Mandatory closure versus nonintervention for patent ductus arteriosus in very preterm infants. J Pediatr. 2016;177:66-71. doi:10.1016/j.jpeds.2016.06.046 27453374

[10] Edstedt Bonamy AK, Gudmundsdottir A, Maier RF, ; Collaborators from the EPICE Research Group. Patent ductus arteriosus treatment in very preterm infants: a European population-based cohort study (EPICE) on variation and outcomes. Neonatology. 2017;111(4):367-375. doi:10.1159/000454798 28125815

[11] Mitra S, Florez ID, Tamayo ME, . Association of placebo, indomethacin, ibuprofen, and acetaminophen with closure of hemodynamically significant patent ductus arteriosus in preterm infants: a systematic review and meta-analysis. JAMA. 2018;319(12):1221-1238. doi:10.1001/jama.2018.1896 29584842 PMC5885871

[12] Sosenko IR, Fajardo MF, Claure N, Bancalari E. Timing of patent ductus arteriosus treatment and respiratory outcome in premature infants: a double-blind randomized controlled trial. J Pediatr. 2012;160(6):929-935. doi:10.1016/j.jpeds.2011.12.031 22284563

[13] Kluckow M, Jeffery M, Gill A, Evans N. A randomised placebo-controlled trial of early treatment of the patent ductus arteriosus. Arch Dis Child Fetal Neonatal Ed. 2014;99(2):F99-F104. doi:10.1136/archdischild-2013-304695 24317704

[14] Clyman RI, Liebowitz M, Kaempf J, ; PDA-TOLERATE (PDA: To Leave It Alone or Respond and Treat Early) Trial Investigators. PDA-TOLERATE trial: an exploratory randomized controlled trial of treatment of moderate-to-large patent ductus arteriosus at 1 week of age. J Pediatr. 2019;205:41-48. doi:10.1016/j.jpeds.2018.09.012 30340932 PMC6502709

[15] Hundscheid T, Onland W, Kooi EMW, ; BeNeDuctus Trial Investigators. Expectant management or early ibuprofen for patent ductus arteriosus. N Engl J Med. 2023;388(11):980-990. doi:10.1056/NEJMoa2207418 36477458

[16] Gupta S, Subhedar NV, Bell JL, ; Baby-OSCAR Collaborative Group. Trial of selective early treatment of patent ductus arteriosus with ibuprofen. N Engl J Med. 2024;390(4):314-325. doi:10.1056/NEJMoa2305582 38265644 PMC7615774

[17] Gould JB, Bennett MV, Phibbs CS, Lee HC. Population improvement bias observed in estimates of the impact of antenatal steroids to outcomes in preterm birth. J Pediatr. 2021;232:17-22. doi:10.1016/j.jpeds.2020.11.067 33275981

[18] Schmidt B, Davis P, Moddemann D, ; Trial of Indomethacin Prophylaxis in Preterms Investigators. Long-term effects of indomethacin prophylaxis in extremely-low-birth-weight infants. N Engl J Med. 2001;344(26):1966-1972. doi:10.1056/NEJM200106283442602 11430325

[19] Jobe AH, Bancalari E. Bronchopulmonary dysplasia. Am J Respir Crit Care Med. 2001;163(7):1723-1729. doi:10.1164/ajrccm.163.7.2011060 11401896

[20] Walsh MC, Yao Q, Gettner P, ; National Institute of Child Health and Human Development Neonatal Research Network. Impact of a physiologic definition on bronchopulmonary dysplasia rates. Pediatrics. 2004;114(5):1305-1311. doi:10.1542/peds.2004-0204 15520112

[21] Higgins JP, Altman DG, Gøtzsche PC, ; Cochrane Bias Methods Group; Cochrane Statistical Methods Group. The Cochrane Collaboration’s tool for assessing risk of bias in randomised trials. BMJ. 2011;343:d5928. doi:10.1136/bmj.d5928 22008217 PMC3196245

[22] Rozé JC, Cambonie G, Le Thuaut A, . Effect of early targeted treatment of ductus arteriosus with ibuprofen on survival without cerebral palsy at 2 years in infants with extreme prematurity: a randomized clinical trial. J Pediatr. 2021;233:33-42. doi:10.1016/j.jpeds.2020.12.008 33307111

[23] El-Khuffash A, Bussmann N, Breatnach CR, . A pilot randomized controlled trial of early targeted patent ductus arteriosus treatment using a risk based severity score (the PDA RCT). J Pediatr. 2021;229:127-133. doi:10.1016/j.jpeds.2020.10.024 33069668

[24] de Waal K, Phad N, Stubbs M, Chen Y, Kluckow M. A randomized placebo-controlled pilot trial of early targeted nonsteroidal anti-inflammatory drugs in preterm infants with a patent ductus arteriosus. J Pediatr. 2021;228:82-86. doi:10.1016/j.jpeds.2020.08.062 32858033

[25] Potsiurko S, Dobryanskyy D, Sekretar L, Salabay Z. Randomized noninferiority trial of expectant management versus early treatment of patent ductus arteriosus in preterm infants. Am J Perinatol. 2024;41(6):730-738. doi:10.1055/a-1782-5860 35213904

[26] Sung SI, Lee MH, Ahn SY, Chang YS, Park WS. Effect of nonintervention vs oral ibuprofen in patent ductus arteriosus in preterm infants: a randomized clinical trial. JAMA Pediatr. 2020;174(8):755-763. doi:10.1001/jamapediatrics.2020.1447 32539121 PMC7296457

[27] Härkin P, Marttila R, Pokka T, Saarela T, Hallman M. Morbidities associated with patent ductus arteriosus in preterm infants: nationwide cohort study. J Matern Fetal Neonatal Med. 2018;31(19):2576-2583. doi:10.1080/14767058.2017.1347921 28651469

[28] Ezenwa B, Pena E, Schlegel A, Bapat R, Shepherd EG, Nelin LD. Effects of practice change on outcomes of extremely preterm infants with patent ductus arteriosus. Acta Paediatr. 2019;108(1):88-93. doi:10.1111/apa.14423 29806710

[29] Laughon MM, Simmons MA, Bose CL. Patency of the ductus arteriosus in the premature infant: is it pathologic? should it be treated? Curr Opin Pediatr. 2004;16(2):146-151. doi:10.1097/00008480-200404000-00005 15021192

[30] Morrow WR, Taylor AF, Kinsella JP, Lally KP, Gerstmann DR, deLemos RA. Effect of ductal patency on organ blood flow and pulmonary function in the preterm baboon with hyaline membrane disease. Crit Care Med. 1995;23(1):179-186. doi:10.1097/00003246-199501000-00028 8001369

[31] Evans N, Kluckow M. Early determinants of right and left ventricular output in ventilated preterm infants. Arch Dis Child Fetal Neonatal Ed. 1996;74(2):F88-F94. doi:10.1136/fn.74.2.F88 8777673 PMC2528520

[32] Taylor AF, Morrow WR, Lally KP, Kinsella JP, Gerstmann DR, deLemos RA. Left ventricular dysfunction following ligation of the ductus arteriosus in the preterm baboon. J Surg Res. 1990;48(6):590-596. doi:10.1016/0022-4804(90)90236-U 2113970

[33] Clyman RI, Mauray F, Heymann MA, Roman C. Cardiovascular effects of patent ductus arteriosus in preterm lambs with respiratory distress. J Pediatr. 1987;111(4):579-587. doi:10.1016/S0022-3476(87)80126-9 3655990

[34] Tooley WH. Hyaline membrane disease: telling it like it was. Am Rev Respir Dis. 1977;115(6 Pt 2):19-28. 326112 10.1164/arrd.1977.115.S.19

[35] McCord JM. Oxygen-derived free radicals in postischemic tissue injury. N Engl J Med. 1985;312(3):159-163. doi:10.1056/NEJM198501173120305 2981404

[36] Eppinger MJ, Deeb GM, Bolling SF, Ward PA. Mediators of ischemia-reperfusion injury of rat lung. Am J Pathol. 1997;150(5):1773-1784. 9137100 PMC1858208

[37] Tsoi SM, Nawaytou H, Almeneisi H, . Prostaglandin-E1 infusion in persistent pulmonary hypertension of the newborn. Pediatr Pulmonol. 2024;59(2):379-388. doi:10.1002/ppul.26759 37975485 PMC10872594

[38] Evans NJ, Archer LN. Doppler assessment of pulmonary artery pressure and extrapulmonary shunting in the acute phase of hyaline membrane disease. Arch Dis Child. 1991;66(1, spec No.):6-11. doi:10.1136/adc.66.1_Spec_No.6 1996896 PMC1590368

[39] Mirza H, Garcia J, McKinley G, . Duration of significant patent ductus arteriosus and bronchopulmonary dysplasia in extremely preterm infants. J Perinatol. 2019;39(12):1648-1655. doi:10.1038/s41372-019-0496-5 31554913

[40] Mitchell MD, Lucas A, Whitfield M, Etches P, Brunt JD, Turnbull AC. Selective elevation of circulating prostaglandin concentrations in hyaline membrane disease in pre-term infants. Prostaglandins Med. 1978;1(3):207-212. doi:10.1016/0161-4630(78)90107-6 715060

[41] Cakir U, Tugcu AU, Tayman C, Yildiz D. Evaluation of the effectiveness of systemic inflammatory indices in the diagnosis of respiratory distress syndrome in preterm with gestational age of ≤32 weeks. Am J Perinatol. 2024;41(suppl 1):e1546-e1552. doi:10.1055/a-2051-8544 36898408

[42] Leroy S, Caumette E, Waddington C, Hébert A, Brant R, Lavoie PM. A time-based analysis of inflammation in infants at risk of bronchopulmonary dysplasia. J Pediatr. 2018;192:60-65. doi:10.1016/j.jpeds.2017.09.011 29092751

[43] Hofstetter AO, Saha S, Siljehav V, Jakobsson PJ, Herlenius E. The induced prostaglandin E_2_ pathway is a key regulator of the respiratory response to infection and hypoxia in neonates. Proc Natl Acad Sci U S A. 2007;104(23):9894-9899. doi:10.1073/pnas.0611468104 17535900 PMC1877988

[44] Hsu HW, Lin TY, Liu YC, Yeh JL, Hsu JH. Molecular mechanisms underlying remodeling of ductus arteriosus: looking beyond the prostaglandin pathway. Int J Mol Sci. 2021;22(6):3238. doi:10.3390/ijms22063238 33810164 PMC8005123

[45] Wei YJ, Hsu R, Lin YC, Wong TW, Kan CD, Wang JN. The association of patent ductus arteriosus with inflammation: a narrative review of the role of inflammatory biomarkers and treatment strategy in premature infants. Int J Mol Sci. 2022;23(22):13877. doi:10.3390/ijms232213877 36430355 PMC9699120

[46] Hammerman C, Strates E, Valaitis S. The silent ductus: its precursors and its aftermath. Pediatr Cardiol. 1986;7(3):121-127. doi:10.1007/BF02424985 3468491

[47] Allegaert K, Anderson B, Simons S, van Overmeire B. Paracetamol to induce ductus arteriosus closure: is it valid? Arch Dis Child. 2013;98(6):462-466. doi:10.1136/archdischild-2013-303688 23606713

[48] Doyle LW, Cheong JL, Hay S, Manley BJ, Halliday HL. Early (<7 days) systemic postnatal corticosteroids for prevention of bronchopulmonary dysplasia in preterm infants. Cochrane Database Syst Rev. 2021;10(10):CD001146. 34674229 10.1002/14651858.CD001146.pub6 PMC8530019

[49] Walters A, McKinlay C, Middleton P, Harding JE, Crowther CA. Repeat doses of prenatal corticosteroids for women at risk of preterm birth for improving neonatal health outcomes. Cochrane Database Syst Rev. 2022;4(4):CD003935. 35377461 10.1002/14651858.CD003935.pub5 PMC8978608

[50] Yeh TF, Chen CM, Wu SY, . Intratracheal administration of budesonide/surfactant to prevent bronchopulmonary dysplasia. Am J Respir Crit Care Med. 2016;193(1):86-95. doi:10.1164/rccm.201505-0861OC 26351971

[51] Pryds O, Greisen G, Johansen KH. Indomethacin and cerebral blood flow in premature infants treated for patent ductus arteriosus. Eur J Pediatr. 1988;147(3):315-316. doi:10.1007/BF00442705 3391227

[52] Edwards AD, Wyatt JS, Richardson C, . Effects of indomethacin on cerebral haemodynamics in very preterm infants. Lancet. 1990;335(8704):1491-1495. doi:10.1016/0140-6736(90)93030-S 1972434

[53] Yaseen H, al Umran K, Ali H, Rustum M, Darwich M, al-Faraidy A. Effects of early indomethacin administration on oxygenation and surfactant requirement in low birth weight infants. J Trop Pediatr. 1997;43(1):42-46. doi:10.1093/tropej/43.1.42 9078828

[54] Janér J, Lassus P, Haglund C, Paavonen K, Alitalo K, Andersson S. Pulmonary vascular endothelial growth factor-C in development and lung injury in preterm infants. Am J Respir Crit Care Med. 2006;174(3):326-330. doi:10.1164/rccm.200508-1291OC 16690974

[55] Schmidt B, Roberts RS, Fanaroff A, ; TIPP Investigators. Indomethacin prophylaxis, patent ductus arteriosus, and the risk of bronchopulmonary dysplasia: further analyses from the Trial of Indomethacin Prophylaxis in Preterms (TIPP). J Pediatr. 2006;148(6):730-734. doi:10.1016/j.jpeds.2006.01.047 16769377

[56] Gournay V, Roze JC, Kuster A, . Prophylactic ibuprofen versus placebo in very premature infants: a randomised, double-blind, placebo-controlled trial. Lancet. 2004;364(9449):1939-1944. doi:10.1016/S0140-6736(04)17476-X 15567009

[57] Amendolia B, Lynn M, Bhat V, Ritz SB, Aghai ZH. Severe pulmonary hypertension with therapeutic l-lysine ibuprofen in 2 preterm neonates. Pediatrics. 2012;129(5):e1360-e1363. doi:10.1542/peds.2011-0117 22492771

[58] Chen X, Han D, Wang X, . Vascular and pulmonary effects of ibuprofen on neonatal lung development. Respir Res. 2023;24(1):39. doi:10.1186/s12931-023-02342-4 36732726 PMC9893598

[59] Wright CJ. Acetaminophen and the developing lung: could there be lifelong consequences? J Pediatr. 2021;235:264-276. doi:10.1016/j.jpeds.2021.02.026 33617854 PMC9810455

[60] Murphy C, Bussmann N, Staunton D, McCallion N, Franklin O, El-Khuffash A. The effect of patent ductus arteriosus treatment with paracetamol on pulmonary vascular resistance. J Perinatol. 2022;42(12):1697-1698. doi:10.1038/s41372-022-01410-9 35585179 PMC9712095

[61] Jensen EA, DeMauro SB, Rysavy MA, ; Eunice Kennedy Shriver National Institute of Child Health and Human Development Neonatal Research Network. Acetaminophen for patent ductus arteriosus and risk of mortality and pulmonary morbidity. Pediatrics. 2024;154(2):e2023065056. doi:10.1542/peds.2023-065056 39011550 PMC11291959

[62] Kluckow M, Evans N. Early echocardiographic prediction of symptomatic patent ductus arteriosus in preterm infants undergoing mechanical ventilation. J Pediatr. 1995;127(5):774-779. doi:10.1016/S0022-3476(95)70172-9 7472835

[63] El-Khuffash A, James AT, Corcoran JD, . A patent ductus arteriosus severity score predicts chronic lung disease or death before discharge. J Pediatr. 2015;167(6):1354-1361. doi:10.1016/j.jpeds.2015.09.028 26474706

[64] Shennan AT, Dunn MS, Ohlsson A, Lennox K, Hoskins EM. Abnormal pulmonary outcomes in premature infants: prediction from oxygen requirement in the neonatal period. Pediatrics. 1988;82(4):527-532. doi:10.1542/peds.82.4.527 3174313

[65] NICHD Neonatal Research Network. Management of the PDA Trial (PDA). ClinicalTrials.gov identifier: NCT03456336. Updated March 21, 2024. Accessed September 8, 2024. https://clinicaltrials.gov/study/NCT03456336

[66] Mitra S, Hébert A, Castaldo M, . Selective early medical treatment of the patent ductus arteriosus in extremely low gestational age infants: a pilot randomised controlled trial protocol (SMART-PDA). BMJ Open. 2024;14(7):e087998. doi:10.1136/bmjopen-2024-087998 39053961 PMC11284877

