# Supplementary material for: Active Treatment vs Expectant Management of Patent Ductus Arteriosus in Preterm Infants: A Meta-Analysis
Source: JAMA Pediatr. 2025 May 27;179(8):877–85. doi: 10.1001/jamapediatrics.2025.1025 (PMC12117495; doi:10.1001/jamapediatrics.2025.1025)
Supplement: Supplement 1. — eTable 1. Search Strategy eTable 2. Excluded Full-Text Articles (n = 24) and Reasons for Their Exclusion eTable 3. Individual Article Characteristics eTable 4. Individual Articles’ Secondary Outcomes and Bronchopulmonary Dysplasia (BPD) Definition eTable 5. Differences Between A Priori PROSPERO Protocol and Final Study eTable 6. Baseline Characteristics eFigure 1. Risk of Bias Assessment eFigure 2. Forest Plots for Primary and Secondary Outcomes Not Demonstrated in the Main Article eFigure 3. Forest Plot Showing Subgroup Analysis at Less Than 29 Weeks for Composite Primary Outcome: Death at 36 Weeks or at Discharge (Whichever Occurred Later) or Moderate to Severe Bronchopulmonary Dysplasia in Figure 2A eFigure 4. Forest Plots of Additional Subgroup Analyses eFigure 5. Forest Plot Showing Death at 36 Weeks eFigure 6. Forest Plot Showing Subgroup Analysis at Less Than 29 Weeks for Outcome: Death at 36 Weeks in eFigure 5 eFigure 7. Forest Plot Showing Subgroup Analysis at Less Than 29 Weeks for Outcome: Death at 36 weeks or at Discharge (Whichever Occurred Later) in Figure 2B eFigure 8. Forest Plot Showing Subgroup Analysis at Less Than 29 Weeks: Moderate to Severe Bronchopulmonary Dysplasia at 36 Weeks in Figure 2C eTable 7. Cause of Death eTable 8. Cause of Death (Grouped by System) eFigure 9. Forest Plot Showing Bronchopulmonary Dysplasia at 36 Weeks eFigure 10. Forest Plot showing Subgroup Analysis at Less Than 29 Weeks for Outcome: Bronchopulmonary Dysplasia (BPD) at 36 Weeks in eFigure 9 eTable 9. Secondary Outcomes for Which Meta-Analysis Was Not Done eReferences [file jamapediatr-e251025-s001.pdf]

Supplemental Online Content

Buvaneswarran S, Wong YL, Liang S, Quek SC, Lee J. Active treatment vs expectant management of patent ductus arteriosus in preterm infants: a meta-analysis. *JAMA Pediatrics*. Published online May 27, 2025. doi:10.1001/jamapediatrics.2025.1025

eTable 1. Search Strategy
eTable 2. Excluded Full-Text Articles (n = 24) and Reasons for Their Exclusion
eTable 3. Individual Article Characteristics
eTable 4. Individual Articles' Secondary Outcomes and Bronchopulmonary Dysplasia (BPD) Definition
eTable 5. Differences Between A Priori PROSPERO Protocol and Final Study
eTable 6. Baseline Characteristics
eFigure 1. Risk of Bias Assessment
eFigure 2. Forest Plots for Primary and Secondary Outcomes Not Demonstrated in the Main Article
eFigure 3. Forest Plot Showing Subgroup Analysis at Less Than 29 Weeks for Composite Primary Outcome: Death at 36 Weeks or at Discharge (Whichever Occurred Later) or Moderate to Severe Bronchopulmonary Dysplasia in Figure 2A
eFigure 4. Forest Plots of Additional Subgroup Analyses
eFigure 5. Forest Plot Showing Death at 36 Weeks
eFigure 6. Forest Plot Showing Subgroup Analysis at Less Than 29 Weeks for Outcome: Death at 36 Weeks in eFigure 5
eFigure 7. Forest Plot Showing Subgroup Analysis at Less Than 29 Weeks for Outcome: Death at 36 weeks or at Discharge (Whichever Occurred Later) in Figure 2B
eFigure 8. Forest Plot Showing Subgroup Analysis at Less Than 29 Weeks: Moderate to Severe Bronchopulmonary Dysplasia at 36 Weeks in Figure 2C
eTable 7. Cause of Death
eTable 8. Cause of Death (Grouped by System)
eFigure 9. Forest Plot Showing Bronchopulmonary Dysplasia at 36 Weeks
eFigure 10. Forest Plot showing Subgroup Analysis at Less Than 29 Weeks for Outcome: Bronchopulmonary Dysplasia (BPD) at 36 Weeks in eFigure 9
eTable 9. Secondary Outcomes for Which Meta-Analysis Was Not Done
eReferences

This supplemental material has been provided by the authors to give readers additional information about their work.

© 2025 Buvaneswarran S et al. *JAMA Pediatrics*.

eTable 1. Search Strategy

PUBMED

No.	Query
**6**	(#1 OR #2) AND #3 AND #5
**5**	"Acetaminophen"[Mesh] OR acetaminophen OR paracetamol OR "Ibuprofen"[Mesh] OR ibuprofen OR brufen OR "Indomethacin"[Mesh] OR indomethacin OR indocid OR "Cardiac Catheterization/methods"[Mesh] OR "Vascular Closure Devices"[Mesh] OR Device closure OR closure OR transcatheter OR Percutaneous OR device OR coil OR catheter OR occlusion OR "Cardiac Surgical Procedures"[Mesh] OR Surgical Ligation OR closure OR ligation OR surgical OR surgery OR clip
**3**	"Ductus Arteriosus"[MeSH Terms] OR "ductus arteriosus, patent"[MeSH Terms] OR "PDA"[All Fields] OR "ductus"[All Fields] OR "ductal"[All Fields] OR "Ductus Arteriosus"[All Fields] OR "arterial duct"[All Fields]
**2**	"Infant, Very Low Birth Weight"[Mesh] OR very low birth weight OR very-low-birth-weight
**1**	"Infant, Extremely Premature"[Mesh] OR Infant, extremely premature OR extremely preterm OR preterm OR infant, newborn OR infan* OR neonat*
Filters: RCT	

EMBASE

No.	Query
**#11**	#8 AND #9 AND [randomized controlled trial]/lim AND [humans]/lim AND [english]/lim AND ([embase]/lim OR [preprint]/lim)
**#10**	#8 AND #9
**#9**	#5 OR #6
**#8**	#3 AND #4 AND #7
**#7**	#1 OR #2
**#6**	lung dysplasia'/exp OR 'bronchopulmonary dysplasia' OR 'chronic lung disease of prematurity'/exp OR 'chronic lung disease of prematurity'
**#5**	death'/exp OR 'death'
**#4**	paracetamol'/exp OR 'paracetamol' OR 'ibuprofen'/exp OR 'ibuprofen' OR 'indomethacin'/exp OR 'indomethacin' OR 'ligation'/exp OR 'ligation' OR 'clip'/exp OR 'clip' OR 'coil'/exp OR 'coil' OR 'device closure'/exp OR 'device closure' OR 'device'/exp OR 'device'
**#3**	patent ductus arteriosus'/exp OR 'patent ductus arteriosus' OR 'ductus'
**#2**	very low birth weight'/exp OR 'very low birth weight'
**#1**	extremely premature birth'/exp OR 'extremely premature birth'

COCHRANE

No.	Query
**#1**	Infant, extremely premature OR extremely preterm OR preterm OR infant, newborn OR infan* OR neonat*
**#2**	very low birth weight OR very-low-birth-weight
**#3**	patent ductus arteriosus OR PDA OR ductus OR ductal OR ductus arteriosus OR arterial duct
**#4**	acetaminophen OR paracetamol OR ibuprofen OR brufen OR indomethacin OR indocid OR Device closure OR closure OR transcatheter OR Percutaneous OR device OR coil OR catheter OR occlusion OR Surgical Ligation OR closure OR ligation OR surgical OR surgery OR clip
**#5**	(#1 OR #2) AND #3 AND #4
**#6**	bronchopulmonary dysplasia OR BPD OR chronic lung disease
**#7**	death OR mortality
**#8**	#5 AND (#6 OR #7) in Trials

© 2025 Buvaneswarran S et al. *JAMA Pediatrics*.

eTable 2. Excluded Full-Text Articles (n = 24) and Reasons for Their Exclusion

**Study Author, Year**	**Reason for Exclusion**
Härkin et al., 2016[1]	No expectant management
Bussmann et al., 2021[2]	Post-hoc analysis of previously reported RCT
Yanagi et al., 1981[3]	No expectant management
Schindler et al., 2021[4]	No expectant management
Kluckow et al., 2019[5]	Post-hoc analysis of previously reported RCT
Ment et al., 1994[6]	No expectant management
Gournay et al., 2004[7]	No expectant management
Rudd et al., 1983[8]	No expectant management
Schmidt et al., 2001[9]	No expectant management
de Carolis et al., 2000[10]	No expectant management
Mahony et al., 1985[11]	No expectant management
Hammerman et al., 2017[12]	No expectant management
Supapannachart et al., 1999[13]	No expectant management
van Overmeire et al., 2004[14]	No expectant management
Aranda et al., 2009[15]	No expectant management
Cassady et al., 1989[16]	No expectant management
Alfaleh et al., 2008[17]	No expectant management
Vincer et al., 1987[18]	No expectant management
Juujärvi et al., 2019[19]	Post-hoc analysis of previously reported RCT
Bandstra et al., 1988[20]	No expectant management
Lai et al., 1990[21]	No expectant management
Couser et al., 1996[22]	No expectant management
Couser et al., 2020[23]	No expectant management
Schmidt et al., 2006[24]	No expectant management

© 2025 Buvaneswarran S et al. *JAMA Pediatrics*.

eTable 3. Individual Article Characteristics

Study, Year	Location (Number of centres)	Study Design	Inclusion	Total Sample Size, n (Study vs. Control)	Active Treatment	Open label medical treatment, n (%) (Study vs. Control)	Surgical ligation, n (%) (Study vs. Control)	Primary Outcome
Sosenko et al., 2012[25]	America (1)	Double-blinded, placebo-controlled RCT	GA 23 - 32 weeks<br>BW 500 – 1250g<br>24HOL – 14 days old<br>Clinical symptoms of hs-PDA with 2DE showing predominant L-R or bidirectional shunt	105 (54 vs 51)	Intravenous Ibuprofen	14 (25.9) vs 23 (45.1)	8 (14.8) vs 10 (19.6)	Number of days of O2 during first 28 days<br>Composite of death by 36wPMA or O2 use at 36wPMA<br>Death by 36wPMA<br>O2 use at 36wPMA<br>Number of days on MV during first 28 days<br>Need for O2 / MV at D28
Kluckow et al., 2014[26]	Australia (3)	Double-blinded, placebo-controlled RCT	GA < 29 weeks<br>2DE before 12HOL showing PDA size > 50% of PNA reference	92 (44 vs 48)	Intravenous Indomethacin	9 (20.5) vs 19 (39.6)	0 (0) vs 2 (4.2)	Composite of death, ≥ Grade II PIVH and cystic PVL by discharge
Clyman 2019[27]	America<br>United Kingdom<br>Sweden<br>Turkey<br>(17)	Placebo-controlled RCT	GA 23-25 weeks and 6-14 DOL<br>GA 26-27 weeks and 8-14 DOL<br>Respiratory support<br>Moderate-large PDA by echocardiographic criteria	202 (104 vs 98)	Intravenous Indomethacin or Ibuprofen or Paracetamol	19 (18.3) vs 47 (48.0)	12 (11.5) vs 12 (12.2)	Need for surgical ligation or PDA follow-up with cardiology after discharge
Hundscheid et al., 2023[28]	Netherlands<br>Belgium<br>Denmark<br>(17)	Non-inferiority RCT	GA < 29 weeks<br>< 72HOL<br>PDA > 1.5mm with L to R predominant shunt	273 (137 vs 136)	Intravenous Ibuprofen	52 (38.0) vs 35 (25.7)	3 (2.2) vs 0 (0)	Composite of NEC IIA and above + moderate-severe BPD + death by 36wPMA

© 2025 Buvaneswarran S et al. *JAMA Pediatrics*.

Gupta et al., 2024[29]	United Kingdom (32)	Double-blinded, placebo-controlled RCT	GA 23 – 28 weeks<br>< 72HOL<br>PDA > 1.5mm with unrestricted L-R flow, no clinical pulmonary hypertension<br>Clinical equipoise	653 (326 vs 327)	Intravenous Ibuprofen	43 (13.2) vs 82 (25.1)	9 (2.8) vs 31 (9.5)	Composite of death by 36wPMA or mod-severe BPD at 36wPMA
Rozé et al., 2021[30]	France (11)	Double-blinded, placebo-controlled RCT	GA 24 – 27 weeks<br>6-12HOL<br>Large PDA calculated using PNA criteria	228 (114 vs 114)	Intravenous Ibuprofen	20 (17.5) vs 71 (62.3)	8 (7.0) vs 15 (1.2)	Survival without CP at PMA 24mth
El-Khuffash et al., 2021[31]	Ireland (1)	Double-blinded, placebo-controlled RCT	GA < 29 weeks<br>36-48HOL<br>Echocardiographic PDA risk score ≥ 5.0	60 (30 vs 30)	Intravenous Ibuprofen	5 (16.7) vs 11 (36.7)	6 (20.0) vs 6 (20.0)	Composite of BPD at 36wPMA and death at discharge
de Waal et al., 2021[32]	Australia (2)	Double-blinded placebo-controlled pilot RCT	GA < 29 weeks<br>< 72HOL<br>PDA > 1.5mm	72 (35 vs 37)	Intravenous Ibuprofen or Indomethacin	1 (2.9) vs 1 (2.7)	0 (0) vs 0 (0)	Recruiting Rate, incidence of open-label treatment
Potsiurko et al., 2024[33]	Ukraine (1)	Non-inferiority RCT	GA <32 weeks<br>BW < 1500g<br>< 72HOL<br>PDA > 1.5mm on 2DE	208 (104 vs 104)	Rectal Ibuprofen or Intravenous Paracetamol	0 (0) vs 8 (7.7)	0 (0) vs 0 (0)	Composite of death by 36wPMA or BPD at 36wPMA
Sung et al., 2020[34]	South Korea (1)	Double-blinded, placebo-controlled RCT	GA 23 – 31 weeks<br>Requiring respiratory support<br>PDA > 1.5mm with predominant L-R shunt at PNA 6 –14 days	142 (70 vs 72)	Oral ibuprofen	1 (1.4) vs 0 (0)	1 (1.4) vs 0 (0)	Composite of death by 36wPMA or BPD at 36wPMA

Abbreviations: BPD, bronchopulmonary dysplasia; BW, body weight; CP, cerebral palsy; DOL, day of life; GA, gestational age; hs-PDA, hemodynamically significant patent ductus arteriosus; HOL, hours of life; L, left; PDA, patent ductus arteriosus; PIVH, periventricular/ intraventricular hemorrhage; PMA, postmenstrual age; PNA, postnatal age; PVL, periventricular leukomalacia; NEC, necrotizing enterocolitis; O2, oxygen; MV, mechanical ventilation; R, right; RCT, randomized controlled trial; 2DE, 2D Echo.

© 2025 Buvaneswarran S et al. *JAMA Pediatrics*.

eTable 4. Individual Articles' Secondary Outcomes and Bronchopulmonary Dysplasia (BPD) Definition

Study, Year	Secondary Outcomes	BPD definition
Sosenko et al., 2012[25]	• Total duration of oxygen supplementation<br>• Duration of mechanical ventilation<br>• Need for oxygen supplementation >30% at 36 weeks<br>• Pneumothorax<br>• Pulmonary interstitial emphysema<br>• Postnatal steroids<br>• Intestinal perforation<br>• NEC requiring surgery<br>• Intracranial hemorrhage<br>• PVL<br>• ROP<br>• Sepsis (defined as positive blood culture results)	Oxygen requirement at 36 weeks' postmenstrual age [Jobe & Bancalari, 2001][35]
Kluckow et al., 2014[26]	• Mortality<br>• Periventricular-IVH<br>• PVL<br>• Total doses and side effects of indomethacin<br>• Renal impairment (elevated creatinine >150 mmol/L, oliguria <1.0 mL/kg/h for >12 h during treatment)<br>• GI bleeding<br>• Spontaneous intestinal perforation<br>• NEC – greater than stage 1 of Bell's classification<br>• Time to full oral feeds (150 mL/kg/day)<br>• PH (frank blood-stained respiratory secretions, a sudden clinical increase in respiratory support and chest x-ray changes)<br>• ROP<br>• Sepsis (positive blood culture and antibiotics >5 days)<br>• Duration of mechanical ventilation, continuous positive airway pressure, or oxygen therapy<br>• Duration of hospitalization<br>• BPD (physiological requirement for oxygen at 36 weeks' GA)<br>• Diuretics or postnatal steroid for BPD<br>• Developmental outcome at 12 and 36 months (planned)	Physiological requirement for oxygen at 36 weeks' GA [Jobe & Bancalari, 2001; Walsh et al., 2004][35,36]
Clyman 2019[27]	• Duration of intubation and respiratory support<br>• Time until achievement of full enteral intake	Modified room air challenge test between 36 0/7 and 36 6/7 weeks post-menstrual age

© 2025 Buvaneswarran S et al. *JAMA Pediatrics*.

	• Duration of gavage feeding<br>• Incidence of serious neonatal morbidities (NEC, BPD, death, and BPD/death)	[Jobe & Bancalari, 2001; Walsh et al., 2004][35,36]
Hundscheid et al., 2023[28]	• Surgical PDA ligation<br>• Death at 28 days<br>• PH<br>• Pulmonary air leakage<br>• Cardiovascular support<br>• Renal failure<br>• IVH<br>• Sepsis<br>• ROP treatment<br>• Co-interventions (steroids, paracetamol, diuretics)<br>• Other support (oxygen, invasive/non-invasive respiratory support)<br>• Time till full enteral feeds	Supplemental oxygen or positive-pressure ventilatory support at 36 weeks postmenstrual age after at least 28 cumulative days of supplemental oxygen, with oxygen reduction test (or expert opinion if not performed)<br>[Jobe & Bancalari, 2001; Walsh et al., 2004][35,36]
Gupta et al., 2024[29]	• Severity of BPD<br>• Severe IVH<br>• Cystic PVL<br>• ROP requiring treatment<br>• Clinically significant PH<br>• Acute pulmonary hypertension<br>• Definitive NEC<br>• Closed or clinically non-significant PDA less than 1.5 mm in diameter with restricted flow at 3 weeks' age<br>• Open-label treatment of a PDA causing symptoms<br>• Weight gain<br>• Discharge home while receiving supplemental oxygen	Supplemental oxygen or positive-pressure ventilatory support at 36 weeks postmenstrual age after at least 28 days of oxygen and/or respiratory support, with oxygen reduction test<br>[Jobe & Bancalari, 2001; Walsh et al., 2004][35,36]
Rozé et al., 2021[30]	• Ductal status on days 3 and 14<br>• Open-label rescue treatment<br>• Surgical ligation<br>• Severe morbidities<br>• Death and survival without severe morbidity at 36 weeks corrected age or discharge, whichever came first<br>• Incidences and duration of mechanical ventilation, non-invasive ventilation and O2 delivery<br>• Ages and Stages Questionnaire (ASQ) total score at 24 months' corrected age	Supplemental oxygen at 36 weeks postmenstrual age, using Walsh's room air challenge test<br>[Jobe & Bancalari, 2001; Walsh et al., 2004][35,36]

© 2025 Buvaneswarran S et al. *JAMA Pediatrics*.

El-Khuffash et al., 2021[31]	• PH<br>• Surgical PDA ligation<br>• Culture positive sepsis<br>• IVH status at 36 weeks corrected age<br>• PVL<br>• NEC<br>• ROP requiring laser therapy<br>• Days spent on invasive ventilation, continuous positive airway pressure, high-flow nasal cannula, and/or oxygen therapy	Oxygen supplementation at 36 weeks corrected age [Shennan et al., 1988][37]
de Waal et al., 2021[32]	• Death or chronic lung disease (CLD), defined as oxygen dependency, determined with an oxygen challenge test at 36 weeks post-conceptual age<br>• Rate of PDA closure, defined as a PDA with a diameter <1.0 mm or closed at 10-14 days post-birth<br>• PDA ligation<br>• PH<br>• IVH<br>• NEC (≥ stage 2b)<br>• GI bleeding<br>• Spontaneous intestinal perforation<br>• Duration of mechanical ventilation<br>• Nasal continuous positive airway pressure<br>• Hospitalization, diuretics, or postnatal steroid use for CLD<br>• ROP necessitating treatment	Oxygen dependency, determined with an oxygen challenge test at 36 weeks post-conceptual age [Jobe & Bancalari, 2001; Walsh et al., 2004][35,36]
Potsiurko et al., 2024[33]	• Mortality<br>• Incidence of BPD<br>• Incidence of NEC (stage Bell's IIA)<br>• PVL<br>• IVH (grade ≥III)<br>• Oliguria (<1 mL/kg/h)<br>• Hypotension (defined as mean blood pressure < gestational age in completed weeks)<br>• Failure to close PDA within a week after the first and second course of pharmacological treatment<br>• Reopening rate of PDA<br>• Need for surgical ligation of PDA<br>• Duration of mechanical ventilation<br>• Duration of noninvasive respiratory support<br>• Duration of oxygen supplementation	Oxygen or respiratory support at 36 weeks postmenstrual age and/or hospital discharge [Jobe & Bancalari, 2001; Walsh et al., 2004][35,36]

© 2025 Buvaneswarran S et al. *JAMA Pediatrics*.

	• Age at administration of full volume of enteral nutrition (160 mL/kg/day)	
Sung et al., 2020[34]	• BPD incidence<br>• Death before discharge<br>• Severe IVH (grade ≥ III)<br>• ROP (stage ≥ 3)<br>• NEC (stage ≥ IIb)<br>• GI surgery<br>• Nosocomial sepsis confirmed on blood culture results	Need for supplemental oxygen and/or positive pressure to maintain oxygen saturation greater than 90% at 36 weeks' GA<br>[Reference not provided]

Abbreviations: GA, gestational age; GI, gastrointestinal; IVH, intraventricular hemorrhage; NEC necrotizing enterocolitis; PDA, patent ductus arteriosus; PH, pulmonary hemorrhage; PVL, periventricular leukomalacia; ROP, retinopathy of prematurity.

© 2025 Buvaneswarran S et al. *JAMA Pediatrics*.

eTable 5. Differences Between A Priori PROSPERO Protocol and Final Study

Included additional primary outcome ("post-hoc outcomes")

1. Death at 36 weeks or at discharge (whichever occurred later).
   Reason for inclusion: Not all included papers reported death at 36 weeks. Some reported death at discharge. Death during hospitalization is a clinically important outcome.
   Inclusion of this outcome resulted in the following additional primary outcomes:
   1) Death at 36 weeks or at discharge (whichever occurred later)
   2) Composite outcome of death at 36 weeks or at discharge (whichever occurred later) or moderate-severe BPD

2. Composite outcome of death at 36 weeks or moderate-severe BPD.
   Reason for inclusion: Clinically important outcome to report.

Additional statistical analysis

1. Included risk difference, in addition to *a priori* plan for risk ratio, in statistical analysis of each primary and secondary outcome.

Abbreviations: BPD, bronchopulmonary dysplasia.

© 2025 Buvaneswarran S et al. *JAMA Pediatrics*.

eTable 6. Baseline Characteristics

**Characteristic**	**Active Treatment (n=1018)**	**Expectant Management (n=1017)**
Male	508/1018 (49.9%)	551/1017 (54.2%)
Gestational Age, weeks*	26.2 ± 1.7	26.3 ± 1.7
Birth Weight, grams*	874.7 ± 222.1	897.7 ± 216.5
Received surfactant	377/464 (81.2%)	374/456 (82.0%)
Multiple births	238/825 (28.8%)	248/825 (30.0%)
Antenatal Steroids	883/1018 (86.7%)	873/1017 (85.8%)
Open label medical treatment	164/1018 (16.1%)	297/1017 (29.2%)

Data are presented as mean±SD or n/n (%).

*Papers that provided median were converted to estimated mean and SD using the DECoMA tool (Chi KY, Li MY, Chen C, Kang E. Cochrane Taiwan. *Data Estimation and Conversion for Meta-Analysis* [DECoMA]. Cochrane Taiwan; 2022. Accessed September 8, 2024. https://taiwan.cochrane.org/resource/decoma).

© 2025 Buvaneswarran S et al. *JAMA Pediatrics*.

eFigure 1. Risk of Bias Assessment

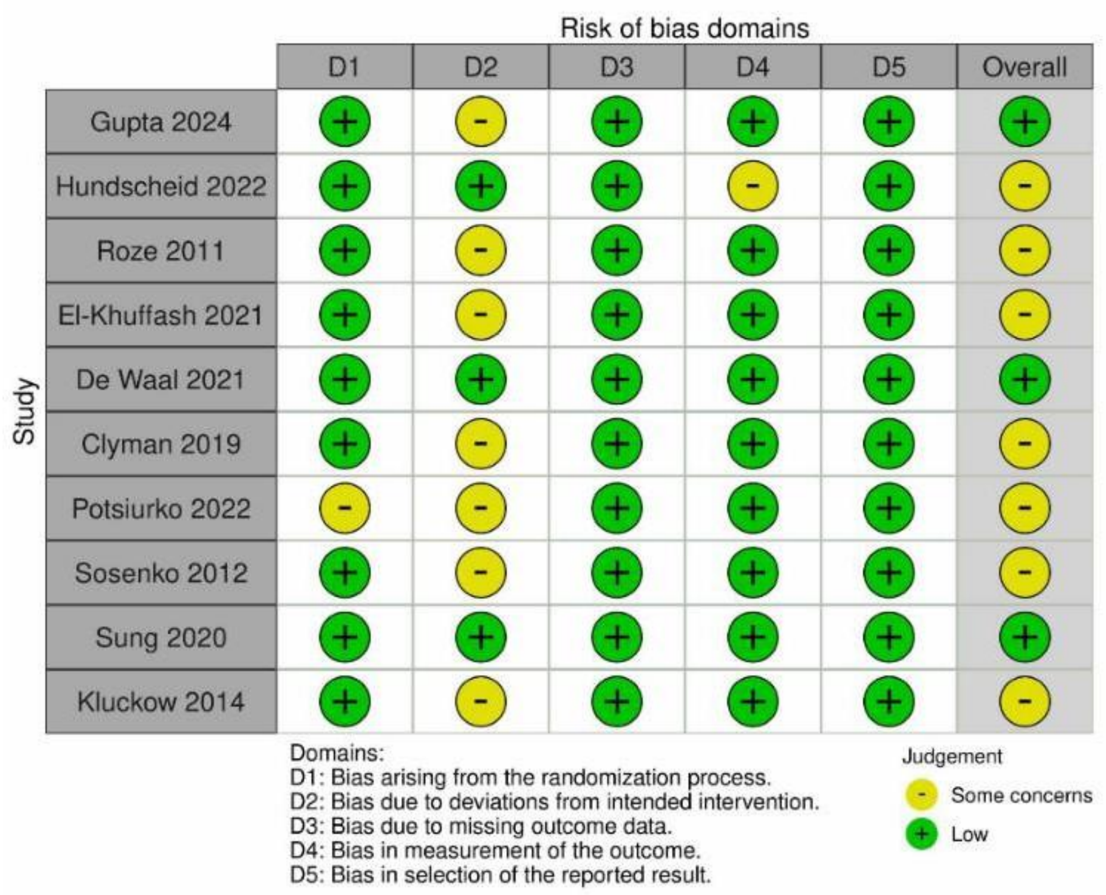

© 2025 Buvaneswarran S et al. *JAMA Pediatrics*.

eFigure 2. Forest Plots for Primary and Secondary Outcomes Not Demonstrated in the Main Article

Death at 36 weeks or moderate/severe BPD at 36 weeks

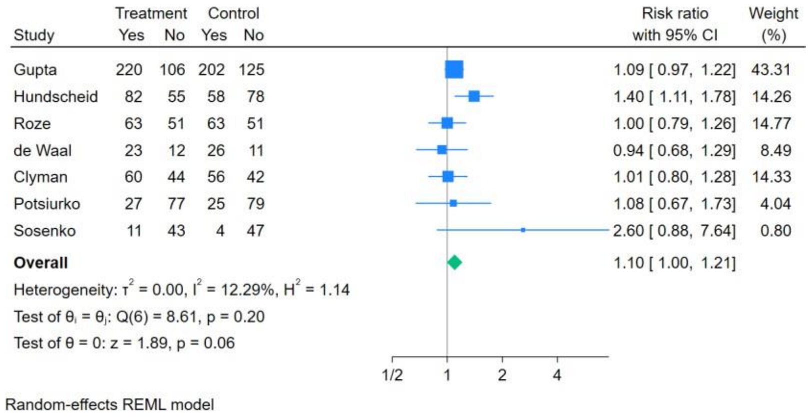

Death before discharge

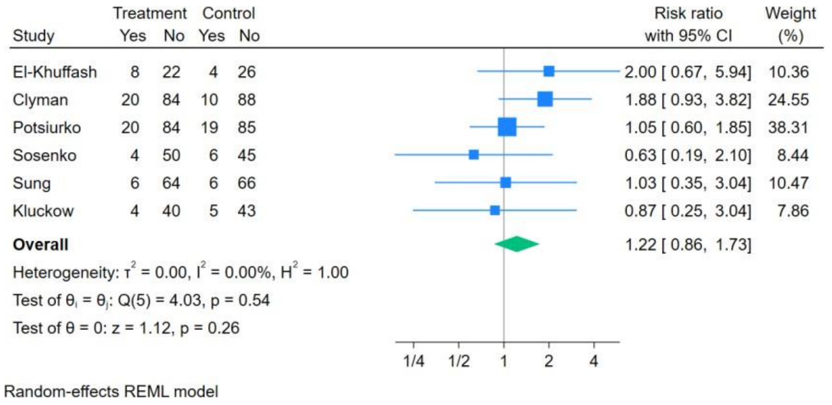

IVH (grade III/IV with ventricular dilation) or intraparenchymal abnormality

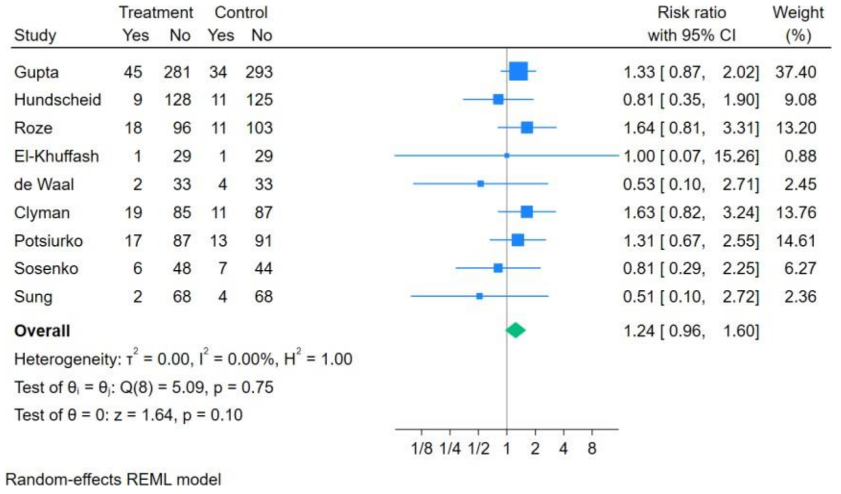

PVL

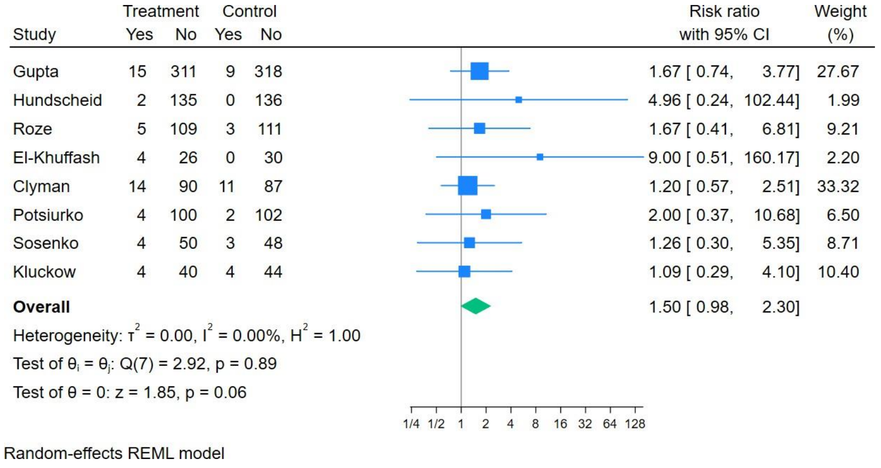

© 2025 Buvaneswarran S et al. *JAMA Pediatrics*.

ROP (stage > 2)

Study	Treatment Yes	Treatment No	Control Yes	Control No	Risk ratio with 95% CI	Weight (%)
Gupta	56	270	54	273	1.04 [0.74, 1.46]	70.44
Roze	3	111	5	109	0.60 [0.15, 2.45]	4.13
Sosenko	7	43	4	46	1.75 [0.55, 5.61]	6.04
Sung	15	55	14	58	1.10 [0.58, 2.11]	19.39
**Overall**					1.06 [0.80, 1.41]	

Heterogeneity: $\tau^2 = 0.00$, $I^2 = 0.00\%$, $H^2 = 1.00$

Test of $\theta_i = \theta_j$: Q(3) = 1.37, p = 0.71

Test of $\theta = 0$: z = 0.41, p = 0.68

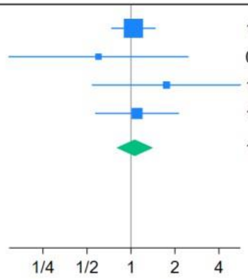

Random-effects REML model

ROP treatment

Study	Treatment Yes	Treatment No	Control Yes	Control No	Risk ratio with 95% CI	Weight (%)
Gupta	45	281	45	282	1.00 [0.68, 1.47]	53.82
Hundscheid	12	99	14	101	0.89 [0.43, 1.83]	15.05
El-Khuffash	4	26	5	25	0.80 [0.24, 2.69]	5.38
de Waal	5	30	2	35	2.64 [0.55, 12.75]	3.20
Clyman	19	85	16	82	1.12 [0.61, 2.05]	21.64
Kluckow	0	44	3	45	0.16 [0.01, 2.93]	0.92
**Overall**					1.01 [0.76, 1.34]	

Heterogeneity: $\tau^2 = 0.00$, $I^2 = 0.00\%$, $H^2 = 1.00$

Test of $\theta_i = \theta_j$: Q(5) = 3.37, p = 0.64

Test of $\theta = 0$: z = 0.07, p = 0.94

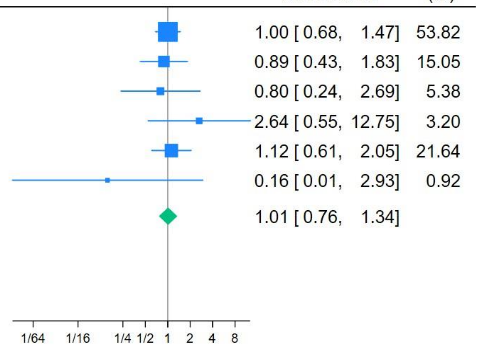

Random-effects REML model

Pulmonary Hemorrhage

Study	Treatment Yes	Treatment No	Control Yes	Control No	Risk ratio with 95% CI	Weight (%)
Gupta	24	302	18	309	1.34 [0.74, 2.42]	24.39
Hundscheid	1	136	4	132	0.25 [0.03, 2.19]	5.50
Roze	5	109	13	101	0.38 [0.14, 1.04]	16.21
de Waal	0	35	1	36	0.35 [0.01, 8.36]	2.85
Clyman	7	97	5	93	1.32 [0.43, 4.02]	14.38
Potsiurko	16	88	11	93	1.45 [0.71, 2.98]	21.60
Kluckow	4	40	11	37	0.40 [0.14, 1.16]	15.07
**Overall**					0.81 [0.46, 1.42]	

Heterogeneity: $\tau^2 = 0.24$, $I^2 = 47.98\%$, $H^2 = 1.92$

Test of $\theta_i = \theta_j$: Q(6) = 10.52, p = 0.10

Test of $\theta = 0$: z = -0.73, p = 0.46

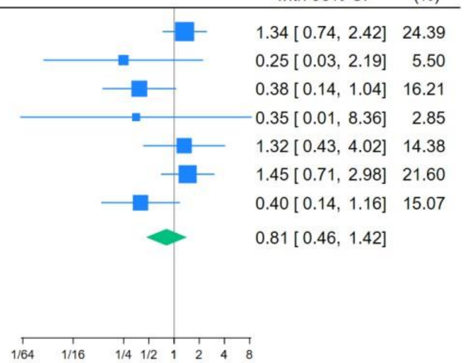

Random-effects REML model

Hypotension OR inotrope/vasopressor OR fluid expansion

Study	Treatment Yes	Treatment No	Control Yes	Control No	Risk ratio with 95% CI	Weight (%)
Gupta	133	193	121	206	1.10 [0.91, 1.34]	44.27
Hundscheid	57	80	60	76	0.94 [0.72, 1.24]	21.86
Roze	55	59	56	58	0.98 [0.75, 1.28]	23.12
El-Khuffash	2	28	2	28	1.00 [0.15, 6.64]	0.46
Clyman	14	90	25	73	0.53 [0.29, 0.96]	4.67
Potsiurko	12	92	12	92	1.00 [0.47, 2.12]	2.90
Kluckow	10	34	10	38	1.09 [0.50, 2.37]	2.73
**Overall**					1.00 [0.88, 1.14]	

Heterogeneity: $\tau^2 = 0.00$, $I^2 = 0.00\%$, $H^2 = 1.00$

Test of $\theta_i = \theta_j$: Q(6) = 5.69, p = 0.46

Test of $\theta = 0$: z = -0.02, p = 0.99

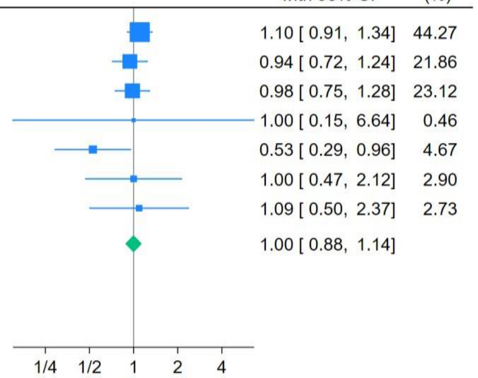

Random-effects REML model

© 2025 Buvaneswarran S et al. *JAMA Pediatrics*.

Kidney Failure

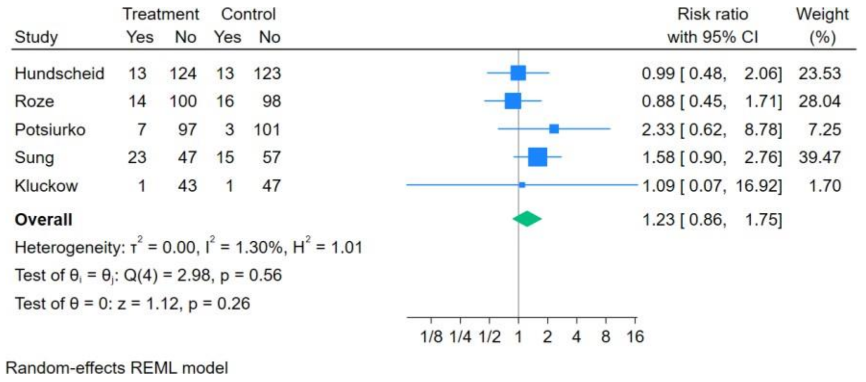

Blood culture positive for sepsis

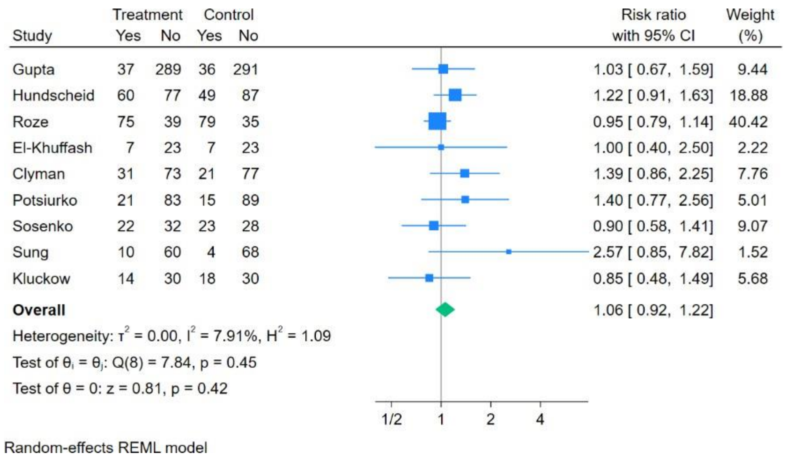

NEC (stage ≥II)

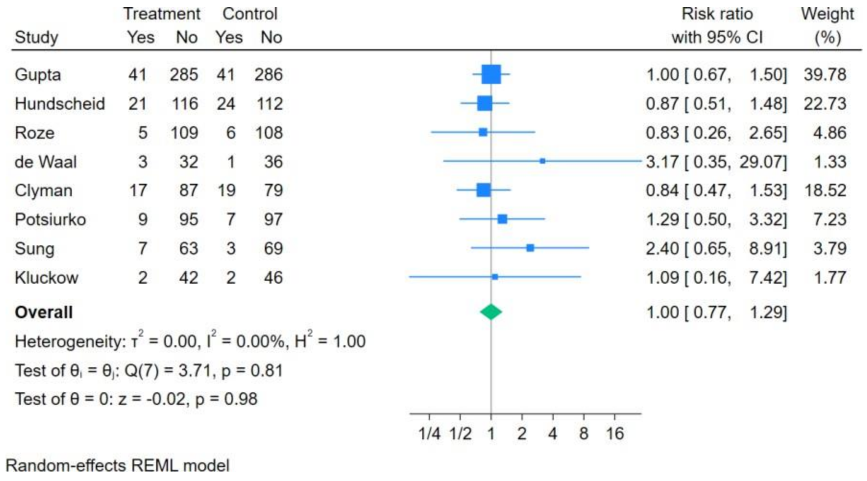

GI perforation

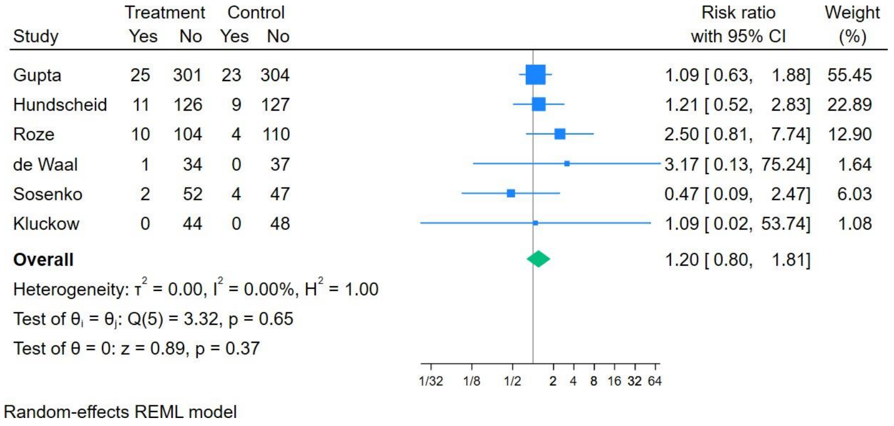

© 2025 Buvaneswarran S et al. *JAMA Pediatrics*.

GI bleed

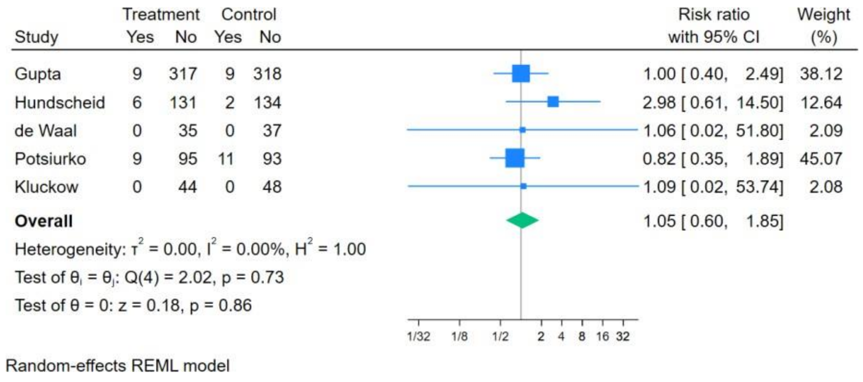

Surgical Ligation

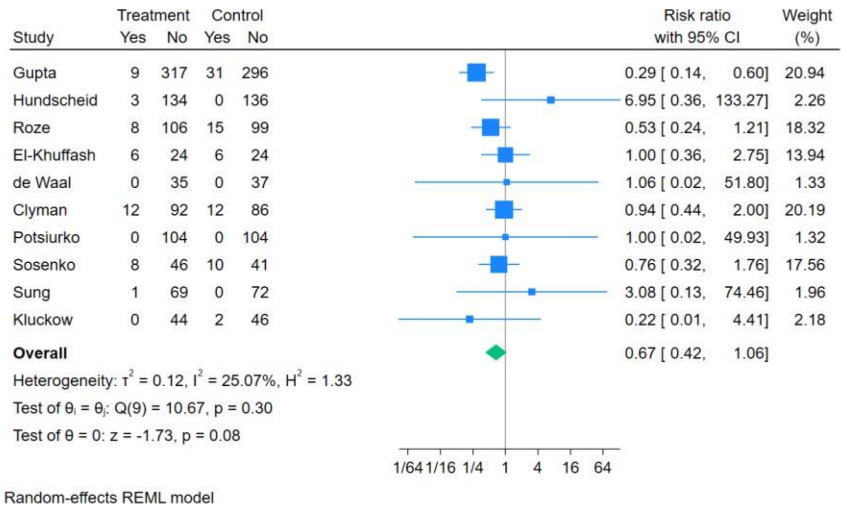

Abbreviations: BPD, bronchopulmonary dysplasia; GI, gastrointestinal; IVH, intraventricular hemorrhage; NEC, necrotizing enterocolitis; PVL, periventricular leukomalacia; ROP, retinopathy of prematurity.

© 2025 Buvaneswarran S et al. *JAMA Pediatrics*.

eFigure 3. Forest Plot Showing Subgroup Analysis at Less Than 29 Weeks for Composite Primary Outcome: Death at 36 Weeks or at Discharge (Whichever Occurred Later) or Moderate to Severe Bronchopulmonary Dysplasia in Figure 2A

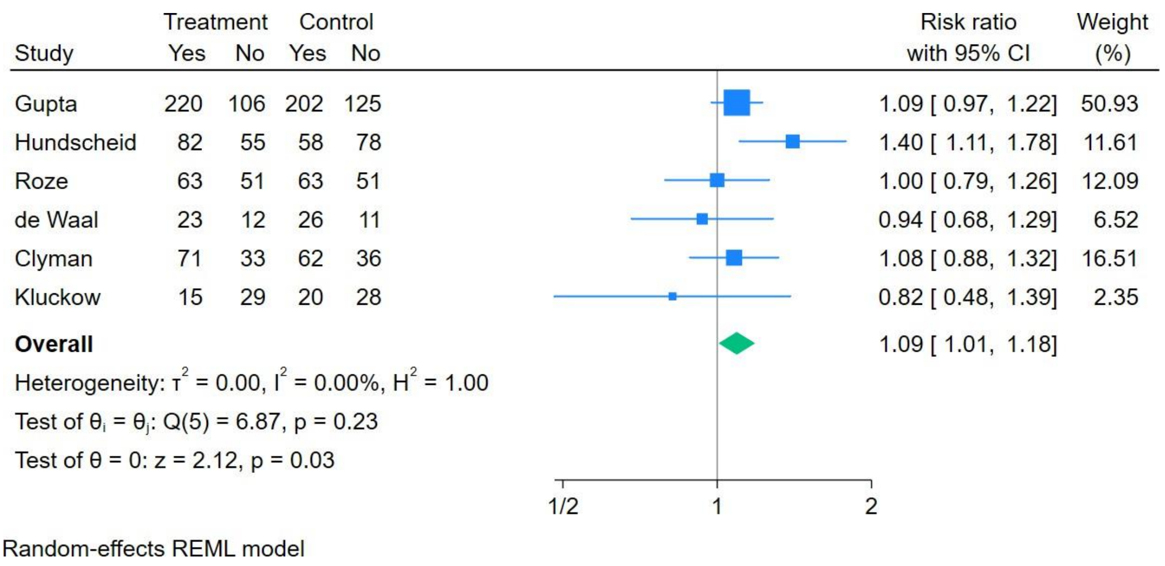

© 2025 Buvaneswarran S et al. *JAMA Pediatrics*.

eFigure 4. Forest Plots of Additional Subgroup Analyses

Death at 36 weeks or at discharge (whichever later) or moderate-severe BPD: Subgroup analysis for <26 weeks

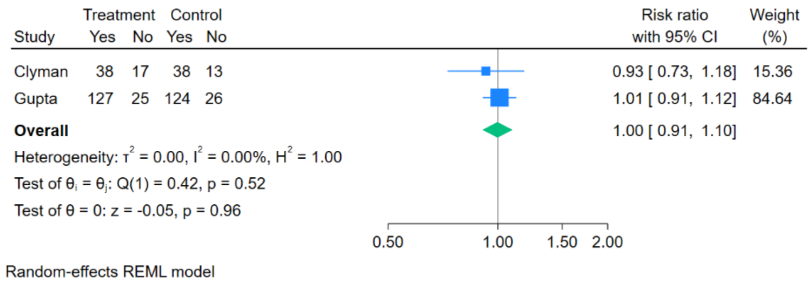

Death at 36 weeks or at discharge (whichever later) or moderate-severe BPD: Subgroup analysis for mode of ventilation at time of randomisation

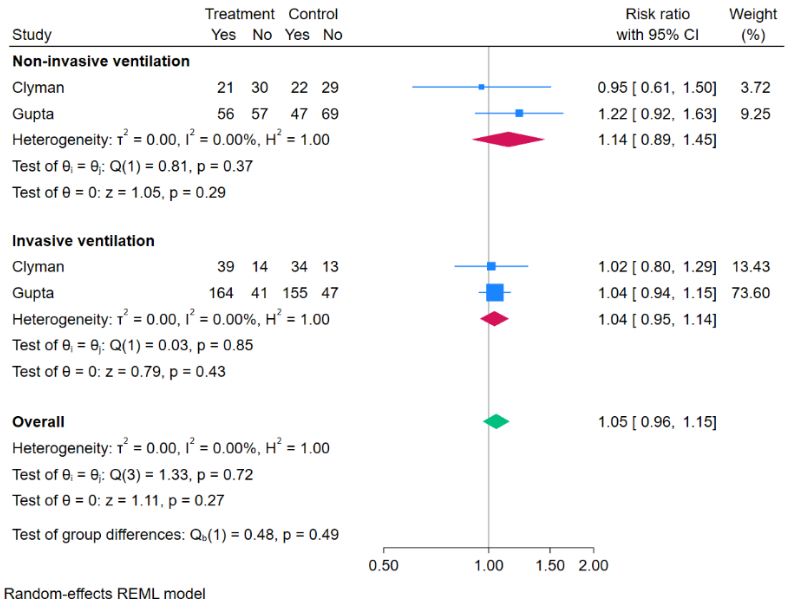

© 2025 Buvaneswarran S et al. *JAMA Pediatrics*.

Death at 36 weeks or at discharge (whichever later) or moderate-severe BPD: Subgroup analysis by hours of life

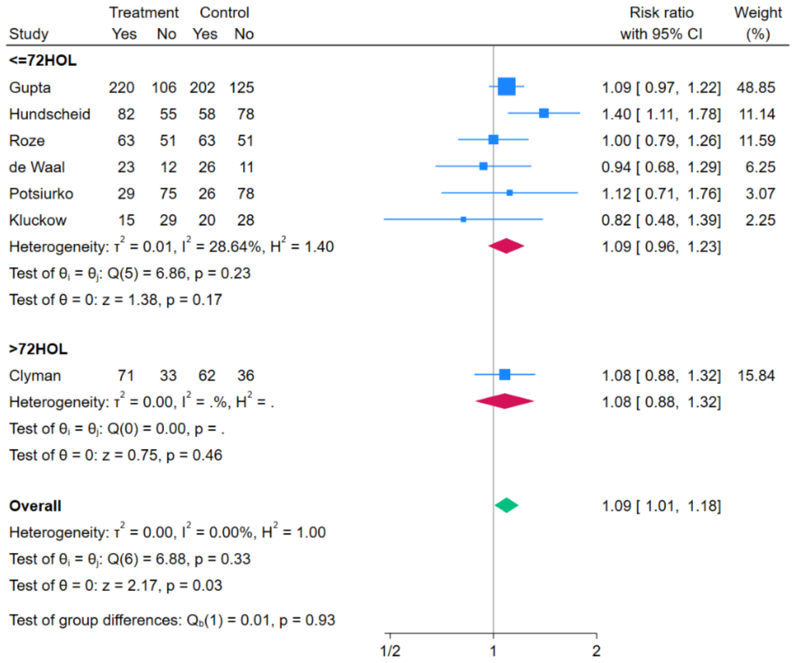

Random-effects REML model

Death at 36 weeks or at discharge (whichever later) or moderate-severe BPD: Subgroup analysis by drug

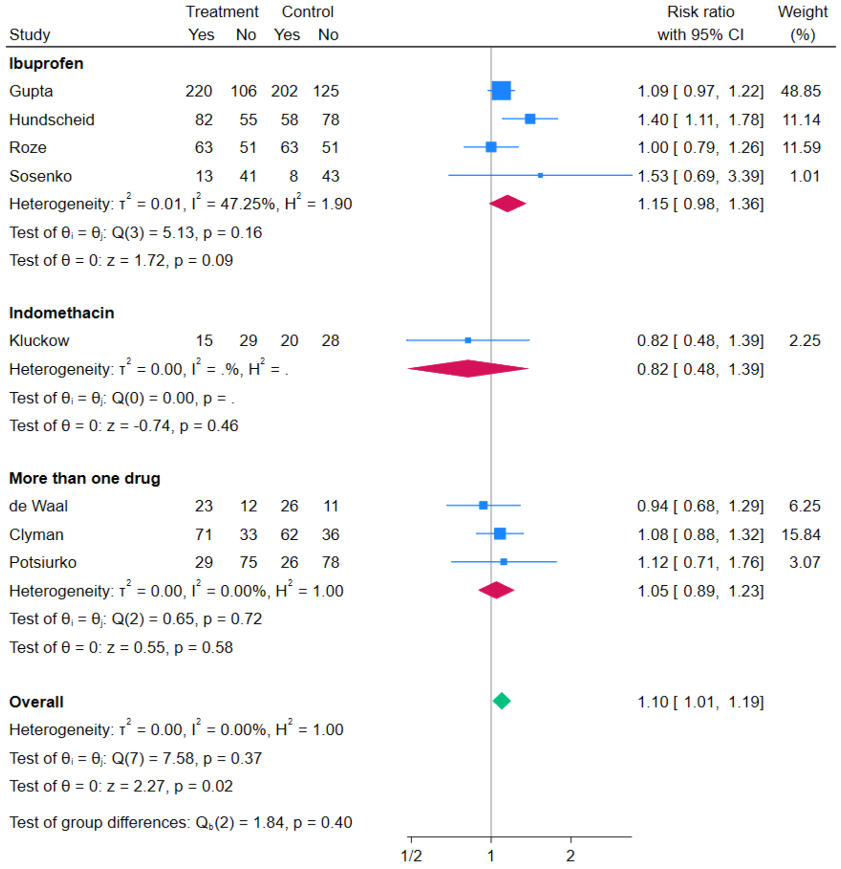

Random-effects REML model

Abbreviations: BPD, bronchopulmonary dysplasia; HOL, hours of life.

© 2025 Buvaneswarran S et al. *JAMA Pediatrics*.

eFigure 5. Forest Plot Showing Death at 36 Weeks

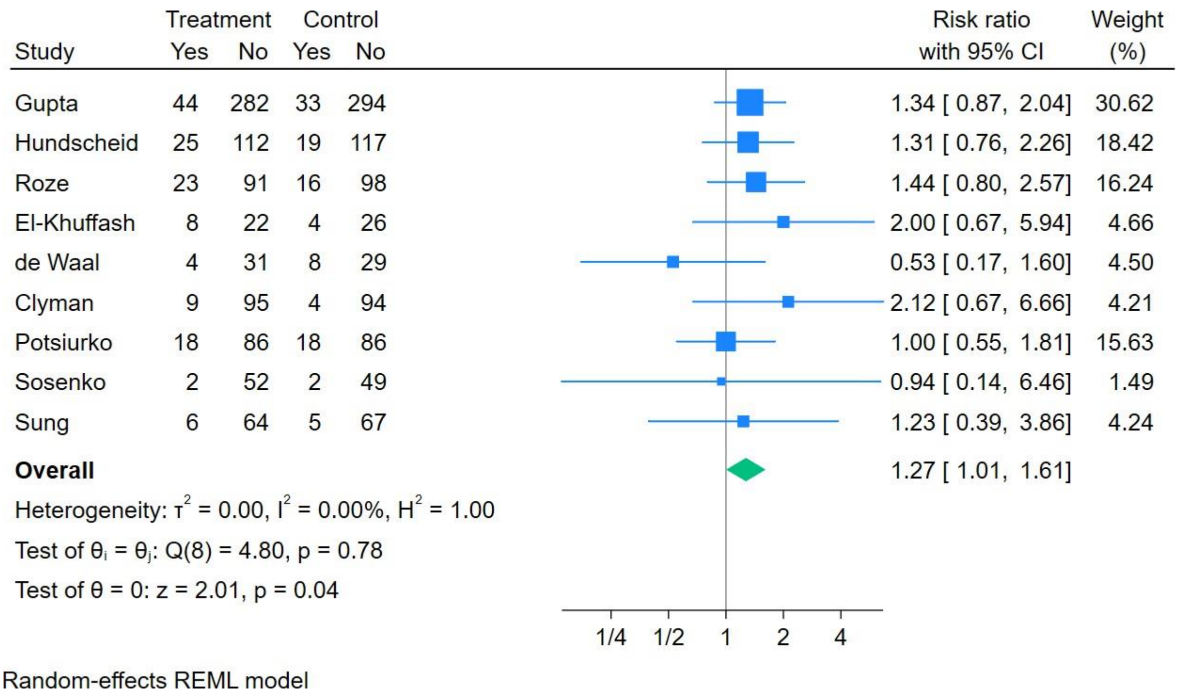

© 2025 Buvaneswarran S et al. *JAMA Pediatrics*.

© 2025 Buvaneswarran S et al. *JAMA Pediatrics*.

eFigure 6. Forest Plot Showing Subgroup Analysis at Less Than 29 Weeks for Outcome: Death at 36 Weeks in eFigure 5

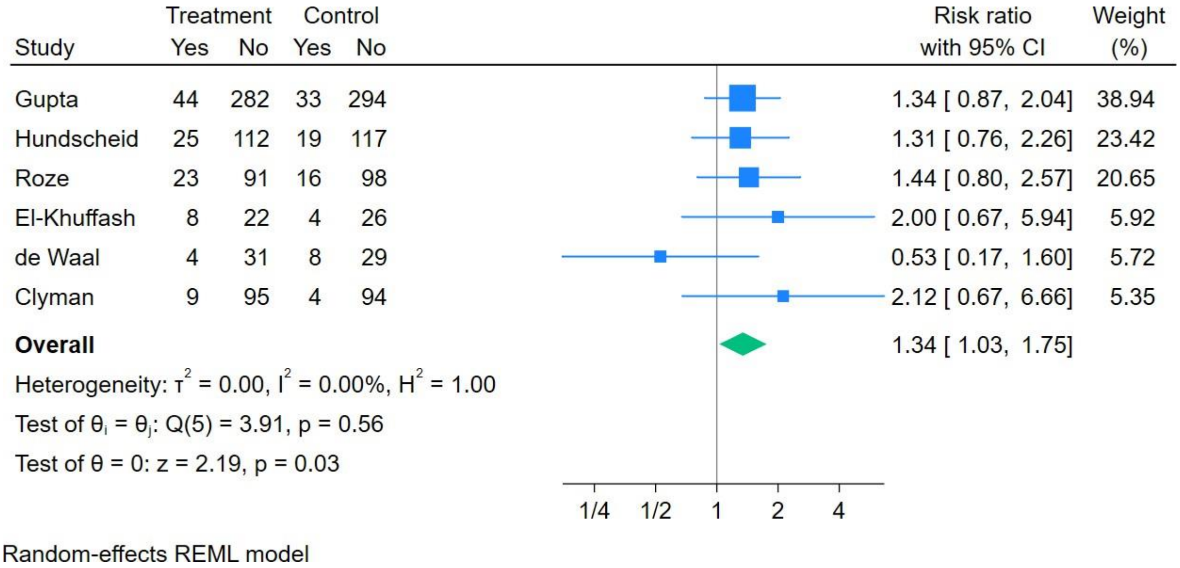

© 2025 Buvaneswarran S et al. *JAMA Pediatrics*.

eFigure 7. Forest Plot Showing Subgroup Analysis at Less Than 29 Weeks for Outcome: Death at 36 Weeks or at Discharge (Whichever Occurred Later) in Figure 2B

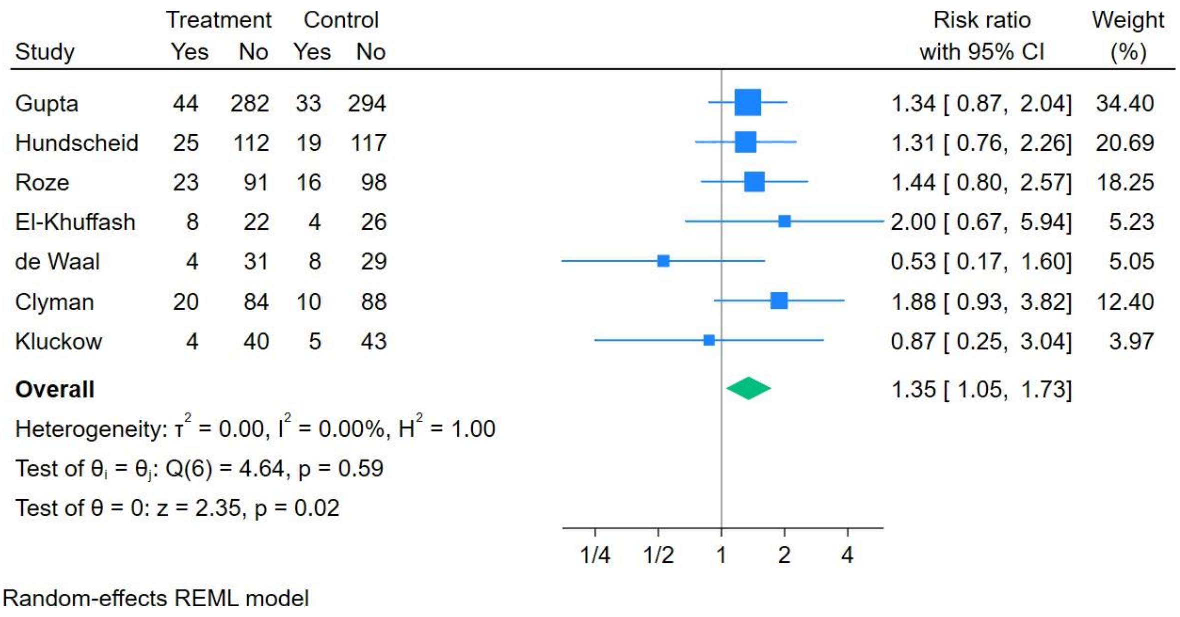

© 2025 Buvaneswarran S et al. *JAMA Pediatrics*.

eFigure 8. Forest Plot Showing Subgroup Analysis at Less Than 29 Weeks: Moderate to Severe Bronchopulmonary Dysplasia at 36 Weeks in Figure 2C

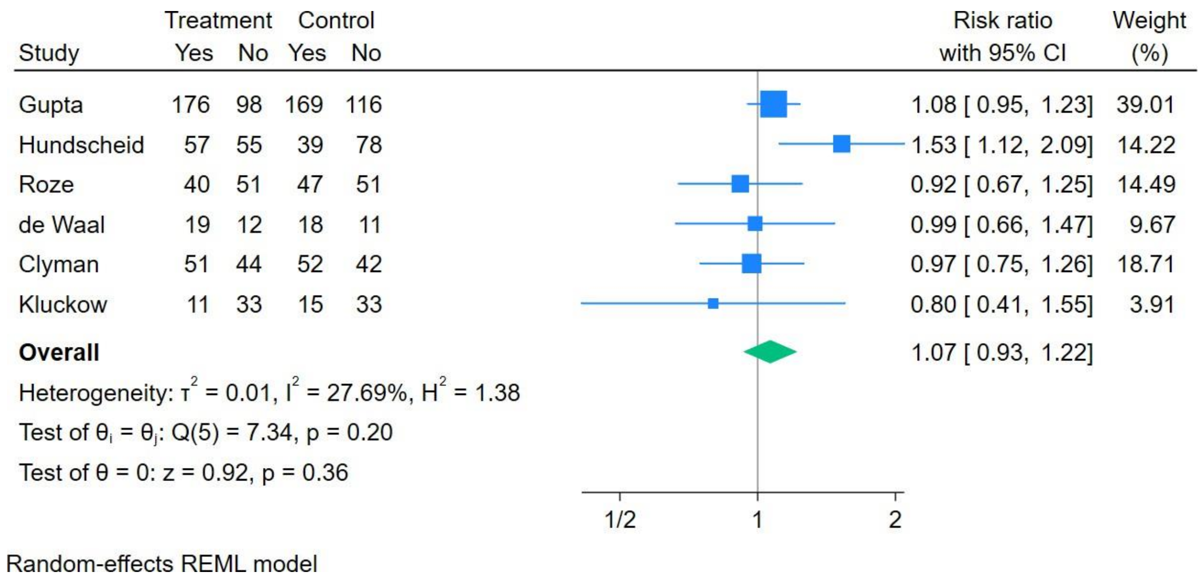

© 2025 Buvaneswarran S et al. *JAMA Pediatrics*.

eTable 7. Cause of Death

S/N	Study, Year		Treatment Arm	Expectant Arm
1	Sosenko et al., 2012[25]	Not available		
2	Kluckow et al., 2014[26]	Not available		
3	Clyman, 2019[27]	Total deaths reported, n/n (%)	20/104 (19.2)	10//98 (10.2)
		BPD, n	0	1
		Intestinal obstruction/volvulus, n	1	1
		NEC, n	9	6
		Bacteremia (non-CoNS)	10	2
4	Hundscheid et al., 2023[28]	Total deaths reported, n/n (%)	25/137 (18.2)	19/136 (14.0)
		Circulatory failure, n	4	1
		Respiratory insufficiency, n	2	2
		NEC/SIP, n	9	7
		Sepsis, n	9	7
		CNS injury, n	1	2
5	Gupta et al., 2024[29]	Not available		
6	Rozé et al., 2021[30]	Not available		
7	El-Khuffash et al., 2021[31]	Total deaths reported, n/n (%)	8/30 (26.7)	4/30 (13·3)
		Respiratory failure/pleural effusion, n	2	2
		NEC, n	3	1
		Sepsis, n	3	0
		IVH, n	0	1
8	de Waal et al., 2021[32]	Total deaths reported, n/n (%)	4/35 (11.4)	8/37 (21.6)
		Respiratory failure	1	2
		IVH, n	1	-
		Sepsis, n	2	3
		NEC, n	-	2
		Kidney and liver failure	-	1
9	Potsiurko et al., 2024[33]	Total deaths reported, n/n (%)	20/104 (19.2)	19/104 (18.3)
		NEC, n	6	3
		CNS, n	11	12
		Severe RDS, n	2	1
		Late-onset sepsis, n	1	3
10	Sung et al., 2020[34]	Not available		
		**Total**	**77**	**60**

Abbreviations: BPD, bronchopulmonary dysplasia; CNS, central nervous system; CoNS, coagulase-negative staphylococci; IVH, intraventricular hemorrhage; NEC, necrotizing enterocolitis; RDS, respiratory distress syndrome; SIP, spontaneous intestinal perforation.

© 2025 Buvaneswarran S et al. *JAMA Pediatrics*.

eTable 8. Cause of Death (Grouped by System)

**Cause of death**	**Treatment Arm**	**Expectant Arm**
Central Nervous System	13	15
Respiratory	11	9
Gastrointestinal	28	20
Sepsis	25	15
Others	0	1
Total	***77***	***60***

© 2025 Buvaneswarran S et al. *JAMA Pediatrics*.

eFigure 9. Forest Plot Showing Bronchopulmonary Dysplasia at 36 Weeks

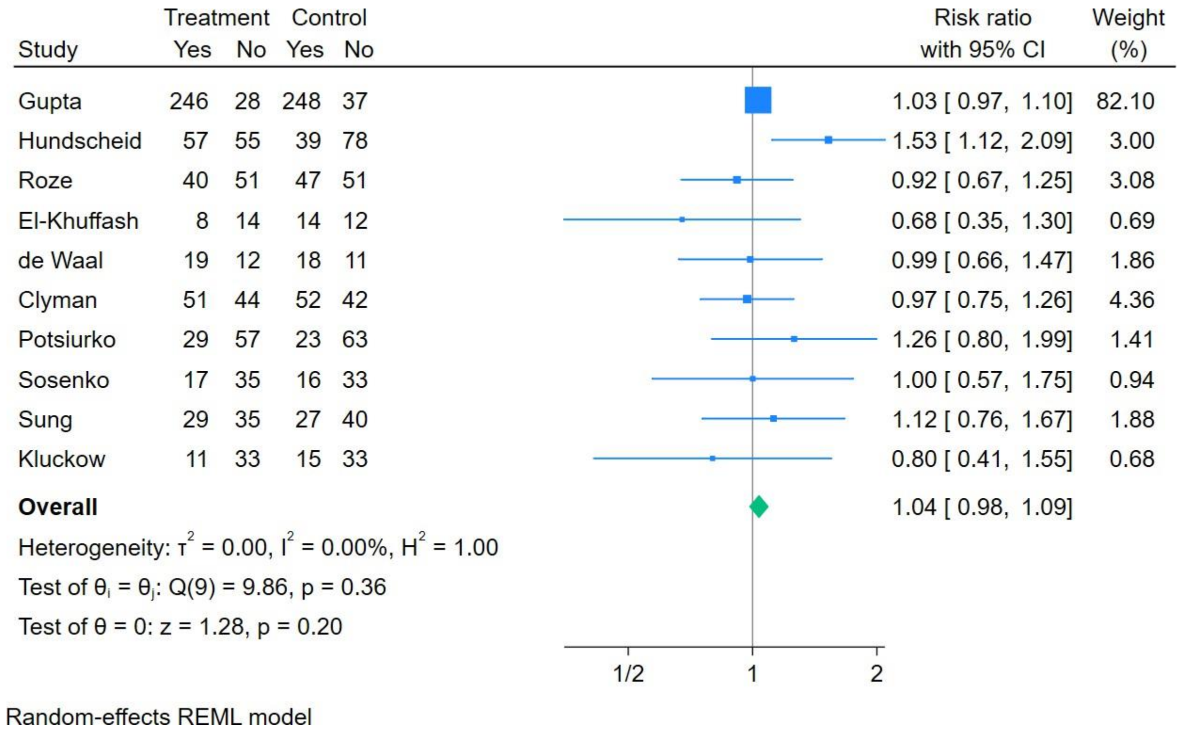

© 2025 Buvaneswarran S et al. *JAMA Pediatrics*.

eFigure 10. Forest Plot showing Subgroup Analysis at Less Than 29 Weeks for Outcome: Bronchopulmonary Dysplasia (BPD) at 36 Weeks in eFigure 9

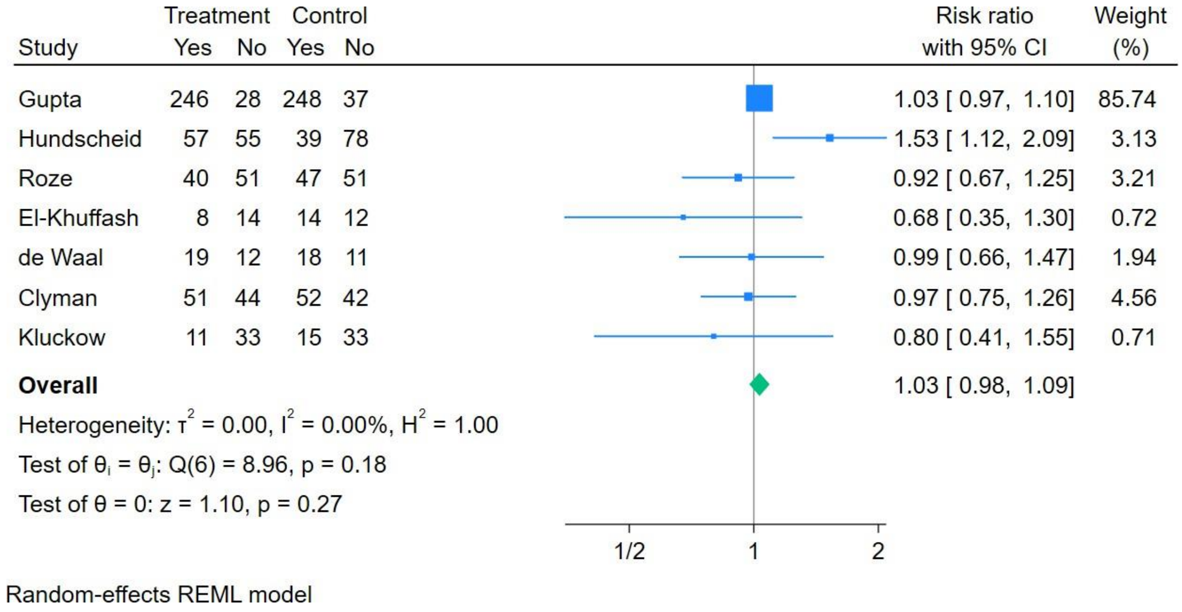

© 2025 Buvaneswarran S et al. *JAMA Pediatrics*.

eTable 9. Secondary Outcomes for Which Meta-Analysis Was Not Done

Secondary Outcome	Result
Cause of Death	Five papers[27,28,31-33] (n=815) reported on cause of death from respiratory insufficiency, sepsis, or necrotising enterocolitis. Four papers (n=613)[28,31-33] reported on death due to central nervous injury. There were no statistically significant differences between the two arms. The cumulative cause of death by category is reported in eTable 8, and by individual paper in eTable 7.
Death at 28 days	One paper[28] (n=273) reported on this outcome. The risk of death at 28 days was increased in the active treatment arm [RR (95% CI): 1.9 (1.02 – 3.57)].
Diuretics	Five papers[27-29,31,32] (n=1260) reported on this outcome. There was less use of diuretics in the active treatment arm compared to the expectant management arm (34.7% vs. 43.3%), which was statistically significant [RR (95% CI): 0.79 (0.69 – 0.91)].
Discharge home on oxygen support	One paper[29] (n=653) reported on this outcome. 39·9% in the active treatment arm vs. 37·6% in the expectant management arm were discharged home on oxygen support. This was not statistically significant [RR (95% CI): 1.06 (0.87 – 1.29)].
Status of PDA at discharge	Two papers[25,33] (n=350) reported that 85.0% in the active treatment arm vs. 79.5% in the expectant management arm had closed PDAs prior to discharge. This was not statistically significant [RR (95% CI): 1.07 (0.97 – 1.18)].<br>Three papers[30,32,33] (n=508) reported on the PDA closure at day 10-14 of life. 166/253 (65.6%) in the active management arm *vs* 111/255 (43.5%) in the expectant management arm achieved this outcome. This was statistically significant [RR (95% CI): 1.51 (1.28 – 1.78)].
Weight gain	One paper[29] (n=653) reported no mean difference in the *Z* scores of weight at birth and at discharge between both arms (mean difference –1 vs. –1.1; standard deviation of 1).
Duration of respiratory support	Six papers[25,28,30,31,33,34] report on duration of oxygen support (medians).<br>Seven papers[25,28-32,34] report on days of invasive respiratory support (medians).<br>Four papers[28-30,34] report on days of non- invasive respiratory support (medians).
Time to full enteral feeding	Five papers[26-28,33,34] report on time to full enteral feeding (medians and one mean).
Duration of hospital stay	Five papers[27,29,31-33] report on duration of hospital stay (medians).

© 2025 Buvaneswarran S et al. *JAMA Pediatrics*.

eReferences

1. Härkin P, Härmä A, Aikio O, et al. Paracetamol accelerates closure of the ductus arteriosus after premature birth: a randomized trial. *J Pediatr.* 2016;177:72-77. doi: 10.1016/j.jpeds.2016.04.066
2. Bussmann N, Smith A, Breatnach CR, et al. Patent ductus arteriosus shunt elimination results in a reduction in adverse outcomes: a post hoc analysis of the PDA RCT cohort. *J Perinatol.* 2021;41(5):1134-1141. doi: 10.1038/s41372-021-01002-z
3. Yanagi RM, Wilson A, Newfeld EA, Aziz KU, Hunt CE. Indomethacin treatment for symptomatic patent ductus arteriosus: a double-blind control study. *Pediatrics*. 1981;67(5):647-652.
4. Schindler T, Smyth J, Bolisetty S, et al. Early PARacetamol (EPAR) trial: a randomized controlled trial of early paracetamol to promote closure of the ductus arteriosus in preterm infants. *Neonatology.* 2021;118(3):274-281. doi: 10.1159/000515415
5. Kluckow M, Carlisle H, Broom M, et al. A pilot randomised blinded placebo-controlled trial of paracetamol for later treatment of a patent ductus arteriosus. *J Perinatol.* 2019;39(1):102-107. doi: 10.1038/s41372-018-0247-z
6. Ment LR, Oh W, Ehrenkranz RA, et al. Low-dose indomethacin and prevention of intraventricular hemorrhage: a multicenter randomized trial. *Pediatrics.* 1994;93(4):543-550.
7. Gournay V, Roze JC, Kuster A, et al. Prophylactic ibuprofen versus placebo in very premature infants: a randomised, double-blind, placebo-controlled trial. *Lancet.* 2004;364(9449):1939-1944. doi: 10.1016/S0140-6736(04)17476-X
8. Rudd P, Montanez P, Hallidie-Smith K, Silverman M. Indomethacin treatment for patent ductus arteriosus in very low birthweight infants: double blind trial. *Arch Dis Child.* 1983;58(4):267-270. doi: 10.1136/adc.58.4.267
9. Schmidt B, Davis P, Moddemann D, et al; Trial of Indomethacin Prophylaxis in Preterms Investigators. Long-term effects of indomethacin prophylaxis in extremely-low-birth-weight infants. *N Engl J Med.* 2001;344(26):1966-1972.
10. De Carolis MP, Romagnoli C, Polimeni V, et al. Prophylactic ibuprofen therapy of patent ductus arteriosus in preterm infants. *Eur J Pediatr.* 2000;159(5):364-368. doi: 10.1007/s004310051288
11. Mahony L, Caldwell RL, Girod DA, et al. Indomethacin therapy on the first day of life in infants with very low birth weight. *J Pediatr.* 1985;106(5):801-805. doi: 10.1016/s0022-3476(85)80361-9
12. Hammerman C, Strates E, Komar K, Bui K. Failure of prophylactic indomethacin to improve the outcome of the very low birth weight infant. *Dev Pharmacol Ther.* 1987;10(6):393-404. doi: 10.1159/000457771

© 2025 Buvaneswarran S et al. *JAMA Pediatrics*.

13. Supapannachart S, Khowsathit P, Patchakapati B. Indomethacin prophylaxis for patent ductus arteriosus (PDA) in infants with a birth weight of less than 1250 grams. *J Med Assoc Thai.* 1999;82 Suppl 1:S87-92.
14. Van Overmeire B, Allegaert K, Casaer A, et al. Prophylactic ibuprofen in premature infants: a multicentre, randomised, double-blind, placebo-controlled trial. *Lancet.* 2004;364(9449):1945-1949. doi: 10.1016/S0140-6736(04)17477-1
15. Aranda JV, Clyman R, Cox B, et al. A randomized, double-blind, placebo-controlled trial on intravenous ibuprofen L-lysine for the early closure of nonsymptomatic patent ductus arteriosus within 72 hours of birth in extremely low-birth-weight infants. *Am J Perinatol.* 2009;26(3):235-245. doi: 10.1055/s-0028-1103515
16. Cassady G, Crouse DT, Kirklin JW, et al. A randomized, controlled trial of very early prophylactic ligation of the ductus arteriosus in babies who weighed 1000 g or less at birth. *N Engl J Med.* 1989;320(23):1511-1516.
17. Alfaleh K, Smyth JA, Roberts RS, et al; Trial of Indomethacin Prophylaxis in Preterms Investigators. Prevention and 18-month outcomes of serious pulmonary hemorrhage in extremely low birth weight infants: results from the trial of indomethacin prophylaxis in preterms. *Pediatrics.* 2008;121(2):e233-e238. doi: 10.1542/peds.2007-0028
18. Vincer M, Allen A, Evans J, et al. Early intravenous indomethacin prolongs respiratory support in very low birth weight infants. *Acta Paediatr Scand.* 1987;76(6):894-897. doi: 10.1111/j.1651-2227.1987.tb17260.x
19. Juujärvi S, Kallankari H, Pätsi P, et al. Follow-up study of the early, randomised paracetamol trial to preterm infants, found no adverse reactions at the two-years corrected age. *Acta Paediatr.* 2019;108(3):452-458. doi: 10.1111/apa.14614
20. Bandstra ES, Montalvo BM, Goldberg RN, et al. Prophylactic indomethacin for prevention of intraventricular hemorrhage in premature infants. *Pediatrics.* 1988;82(4):533-542.
21. Lai TH, Soong WJ, Hwang B. Indomethacin for the prevention of symptomatic patent ductus arteriosus in very low birth weight infants. *Zhonghua Min Guo Xiao Er Ke Yi Xue Hui Za Zhi.* 1990;31(1):17-23.
22. Couser RJ, Ferrara TB, Wright GB, et al. Prophylactic indomethacin therapy in the first twenty-four hours of life for the prevention of patent ductus arteriosus in preterm infants treated prophylactically with surfactant in the delivery room. *J Pediatr.* 1996;128(5 Pt 1):631-637. doi: 10.1016/s0022-3476(96)80127-2
23. Couser RJ, Hoekstra RE, Ferrara TB, Wright GB, Cabalka AK, Connett JE. Neurodevelopmental follow-up at 36 months' corrected age of preterm infants treated with prophylactic indomethacin. *Arch Pediatr Adolesc Med.* 2000;154(6):598-602. doi: 10.1001/archpedi.154.6.598
24. Schmidt B, Roberts RS, Fanaroff A, et al; TIPP Investigators. Indomethacin prophylaxis, patent ductus arteriosus, and the risk of bronchopulmonary dysplasia: further analyses from the Trial of

© 2025 Buvaneswarran S et al. *JAMA Pediatrics*.

Indomethacin Prophylaxis in Preterms (TIPP). *J Pediatr.* 2006;148(6):730-734. doi: 10.1016/j.jpeds.2006.01.047

25. Sosenko IR, Fajardo MF, Claure N, Bancalari E. Timing of patent ductus arteriosus treatment and respiratory outcome in premature infants: a double-blind randomized controlled trial. *J Pediatr.* 2012;160(6):929-935. doi:10.1016/j.jpeds.2011.12.031
26. Kluckow M, Jeffery M, Gill A, Evans N. A randomised placebo-controlled trial of early treatment of the patent ductus arteriosus. *Arch Dis Child Fetal Neonatal Ed.* 2014;99(2):F99-F104. doi:10.1136/archdischild-2013-304695
27. Clyman RI, Liebowitz M, Kaempf J, et al; PDA-TOLERATE (PDA: To Leave It Alone or Respond and Treat Early) Trial Investigators. PDA-TOLERATE trial: an exploratory randomized controlled trial of treatment of moderate-to-large patent ductus arteriosus at 1 week of age. *J Pediatr.* 2019;205:41-48.e6. doi:10.1016/j.jpeds.2018.09.012
28. Hundscheid T, Onland W, Kooi EMW, et al; BeNeDuctus Trial Investigators. Expectant management or early ibuprofen for patent ductus arteriosus. *N Engl J Med.* 2023;388(11):980-990. doi:10.1056/NEJMoa2207418
29. Gupta S, Subhedar NV, Bell JL, et al; Baby-OSCAR Collaborative Group. Trial of selective early treatment of patent ductus arteriosus with ibuprofen. *N Engl J Med.* 2024;390(4):314-325. doi:10.1056/NEJMoa2305582
30. Rozé JC, Cambonie G, Le Thuaut A, et al. Effect of early targeted treatment of ductus arteriosus with ibuprofen on survival without cerebral palsy at 2 years in infants with extreme prematurity: a randomized clinical trial. *J Pediatr.* 2021;233:33-42. doi:10.1016/j.jpeds.2020.12.008
31. El-Khuffash A, Bussmann N, Breatnach CR, et al. A pilot randomized controlled trial of early targeted patent ductus arteriosus treatment using a risk based severity score (the PDA RCT). *J Pediatr.* 2021;229:127-133. doi:10.1016/j.jpeds.2020.10.024
32. de Waal K, Phad N, Stubbs M, Chen Y, Kluckow M. A Randomized placebo-controlled pilot trial of early targeted nonsteroidal anti-inflammatory drugs in preterm infants with a patent ductus arteriosus. *J Pediatr.* 2021;228:82-86. doi:10.1016/j.jpeds.2020.08.062
33. Potsiurko S, Dobryanskyy D, Sekretar L, Salabay Z. Randomized noninferiority trial of expectant management versus early treatment of patent ductus arteriosus in preterm infants. *Am J Perinatol*. 2024;41(6):730-738. doi:10.1055/a-1782-5860
34. Sung SI, Lee MH, Ahn SY, Chang YS, Park WS. Effect of nonintervention vs oral ibuprofen in patent ductus arteriosus in preterm infants: a randomized clinical trial. *JAMA Pediatr.* 2020;174(8):755-763. doi:10.1001/jamapediatrics.2020.1447
35. Jobe AH, Bancalari E. Bronchopulmonary dysplasia. *Am J Respir Crit Care Med.* 2001;163(7):1723-1729. doi:10.1164/ajrccm.163.7.2011060

© 2025 Buvaneswarran S et al. *JAMA Pediatrics*.

36. Walsh MC, Yao Q, Gettner P, et al: National Institute of Child Health and Human Development Neonatal Research Network. Impact of a physiologic definition on bronchopulmonary dysplasia rates. *Pediatrics.* 2004;114(5):1305-1311. doi: 10.1542/peds.2004-0204
37. Shennan AT, Dunn MS, Ohlsson A, Lennox K, Hoskins EM. Abnormal pulmonary outcomes in premature infants: prediction from oxygen requirement in the neonatal period. *Pediatrics.* 1988;82(4):527-532. doi: 10.1542/peds.82.4.527

© 2025 Buvaneswarran S et al. *JAMA Pediatrics*.
