# Supplementary material for: Active Treatment vs Expectant Management of Patent Ductus Arteriosus in Preterm Infants: A Meta-Analysis
Source: JAMA Pediatr. 2025 May 27;179(8):877–85. doi: 10.1001/jamapediatrics.2025.1025 (PMC12117495; doi:10.1001/jamapediatrics.2025.1025)
Supplement: Supplement 2. — Data Sharing Statement [file jamapediatr-e251025-s002.pdf]

Data Sharing Statement

Buvaneswarran. Active Treatment vs Expectant Management of Patent Ductus Arteriosus in Preterm Infants. *JAMA Pediatr*. Published May 27, 2025.
doi:10.1001/jamapediatrics.2025.1025

Data
Data available: Yes
Data types: Other (please specify)
Additional Information: This meta-analysis did not require the collection of new data, but rather the analysis of previously published data.
How to access data: The corresponding author, JL, had full access to all the data in the study, and takes responsibility for the integrity of the data and the accuracy of the data analysis.
When available: With publication

Supporting Documents
Document types: None

Additional Information
Who can access the data: Anyone requesting the meta-analysis process
Types of analyses: For any purpose
Mechanisms of data availability: With investigator support
Any additional restrictions: Nil
